# MiR-9 is overexpressed in spontaneous canine osteosarcoma and promotes a metastatic phenotype including invasion and migration in osteoblasts and osteosarcoma cell lines

**DOI:** 10.1186/s12885-016-2837-5

**Published:** 2016-10-10

**Authors:** Joelle M. Fenger, Ryan D. Roberts, O. Hans Iwenofu, Misty D. Bear, Xiaoli Zhang, Jason I. Couto, Jaime F. Modiano, William C. Kisseberth, Cheryl A. London

**Affiliations:** 1 Department of Veterinary Clinical Sciences, College of Veterinary Medicine, The Ohio State University, 601 Vernon L. Tharp Street, Columbus, OH USA; 2 Center for Childhood Cancer, Nationwide Children’s Hospital, 700 Children’s Drive, Columbus, OH USA; 3 Department of Pathology, College of Medicine, The Ohio State University, 129 Hamilton Hall, 1645 Neil Avenue, Columbus, OH USA; 4 Department of Veterinary Biosciences, College of Veterinary Medicine, The Ohio State University, 1900 Coffey Road, Columbus, OH USA; 5 Center for Biostatistics, The Ohio State University, 320B Lincoln Tower, 1800 Cannon Drive, Columbus, OH USA; 6 Department of Veterinary Clinical Sciences, College of Veterinary Medicine, University of Minnesota, Saint Paul, MN USA; 7 Masonic Cancer Center, University of Minnesota, 420 Delaware Street, SE, MMC 806, Minneapolis, MN USA; 8 444 Veterinary Medical Academic Building, 1600 Coffey Road, Columbus, OH 43210 USA

**Keywords:** MicroRNA, miR-9, Osteosarcoma, Canine, Comparative oncology

## Abstract

**Background:**

MicroRNAs (miRNAs) regulate the expression of networks of genes and their dysregulation is well documented in human malignancies; however, limited information exists regarding the impact of miRNAs on the development and progression of osteosarcoma (OS). Canine OS exhibits clinical and molecular features that closely resemble the corresponding human disease and it is considered a well-established spontaneous animal model to study OS biology. The purpose of this study was to investigate miRNA dysregulation in canine OS.

**Methods:**

We evaluated miRNA expression in primary canine OS tumors and normal canine osteoblast cells using the nanoString nCounter system. Quantitative PCR was used to validate the nanoString findings and to assess miR-9 expression in canine OS tumors, OS cell lines, and normal osteoblasts. Canine osteoblasts and OS cell lines were stably transduced with pre-miR-9 or anti-miR-9 lentiviral constructs to determine the consequences of miR-9 on cell proliferation, apoptosis, invasion and migration. Proteomic and gene expression profiling of normal canine osteoblasts with enforced miR-9 expression was performed using 2D-DIGE/tandem mass spectrometry and RNA sequencing and changes in protein and mRNA expression were validated with Western blotting and quantitative PCR. OS cell lines were transduced with gelsolin (GSN) shRNAs to investigate the impact of GSN knockdown on OS cell invasion.

**Results:**

We identified a unique miRNA signature associated with primary canine OS and identified miR-9 as being significantly overexpressed in canine OS tumors and cell lines compared to normal osteoblasts. Additionally, high miR-9 expression was demonstrated in tumor-specific tissue obtained from primary OS tumors. In normal osteoblasts and OS cell lines transduced with miR-9 lentivirus, enhanced invasion and migration were observed, but miR-9 did not affect cell proliferation or apoptosis. Proteomic and transcriptional profiling of normal canine osteoblasts overexpressing miR-9 identified alterations in numerous genes, including upregulation of GSN, an actin filament-severing protein involved in cytoskeletal remodeling. Lastly, stable downregulation of miR-9 in OS cell lines reduced GSN expression with a concomitant decrease in cell invasion and migration; concordantly, cells transduced with GSN shRNA demonstrated decreased invasive properties.

**Conclusions:**

Our findings demonstrate that miR-9 promotes a metastatic phenotype in normal canine osteoblasts and malignant OS cell lines, and that this is mediated in part by enhanced GSN expression. As such, miR-9 represents a novel target for therapeutic intervention in OS.

**Electronic supplementary material:**

The online version of this article (doi:10.1186/s12885-016-2837-5) contains supplementary material, which is available to authorized users.

## Background

Osteosarcoma (OS) is the most common form of malignant bone cancer in dogs and children, although the incidence of disease in the canine population is approximately ten times higher than in people [1–3]. Both clinical and molecular evidence suggest that human and canine OS share many key features, including anatomic location, presence of microscopic metastatic disease at diagnosis, development of chemotherapy-resistant metastases, altered expression/activation of several proteins (e.g. Met, PTEN, STAT3), and p53 inactivation, among others [2, 3]. Additionally, canine and pediatric OS exhibit overlapping transcriptional profiles and shared DNA copy number aberrations, supporting the notion that these diseases possess significant similarity at the molecular level [4–7]. A defining feature of OS in both species is the high rate of early microscopic metastatic disease. The adoption of multidrug chemotherapy protocols and aggressive surgical techniques has improved survival; however, approximately 30 % of children and over 90 % of dogs ultimately die from metastasis and there has been no significant improvement in clinical outcome in both species over the past 20 years [3, 8].

MicroRNAs (miRNAs) are small non-coding RNAs that negatively regulate gene expression at the post-transcriptional level, resulting in either mRNA cleavage and/or translational repression. Their functions extend to both physiological and pathological conditions, including cell fate specification, cell death, development, metabolism, and cancer [9, 10]. Aberrant miRNA expression is commonly associated with human cancers and it is well established that miRNAs can play a causal role in tumorigenesis, functioning as tumor suppressors or oncogenes by targeting genes involved in tumor development, progression or metastasis [11, 12]. As miRNAs can affect multiple genes in a molecular pathway, or within the context of a network, they likely regulate many distinct biological processes relevant to normal and malignant cell homeostasis [13, 14]. Furthermore, experimental data demonstrate that targeting miRNA expression using chemically modified oligonucleotides can efficiently block the function of miRNAs deregulated in malignant cells and alter cancer phenotypes, establishing the rationale for targeting miRNAs therapeutically in some cancers [15–17]. A variety of miRNA formulations and target-specific delivery strategies have accelerated the clinical development of antisense miRNAs (antago-miRs) or miRNA mimics, several of which have entered human clinical trials. For example, Miravirsen (Santaris Pharma) and MRX34 (Mirna Therapeutics) are being evaluated in patients with chronic hepatitis C virus infection, primary liver cancer, and metastatic cancer that has spread to the liver [18, 19].

Altered miRNA expression profiles have been identified in human OS and unique miRNA signatures are associated with risk of metastasis and response to chemotherapy in this disease [20–27]. Studies evaluating miRNA dysregulation in naturally occurring canine cancers demonstrate that similar to their human counterpart, aberrant miRNA expression likely contributes to tumor biology, although few studies have investigated their contribution to canine OS [28–31]. In human OS, dysregulated miRNAs have been shown to play a direct role in promoting cell proliferation, evading apoptosis, and enhancing motility and invasion. For example, decreased expression of miR-183 in human OS tissues correlates with lung metastasis and local recurrence, in part due to targeting of the membrane-cytoskeleton linker ezrin by miR-183 [32–34]. MiR-125b is frequently down-regulated in human OS tumors and OS cell lines and promotes OS cell proliferation and migration in vitro and tumor formation in vivo by regulating expression of the functional downstream target STAT3 [35].

Recent work has demonstrated down-regulation of a large number of miRNAs at the 14q32 locus in human OS tumors compared to normal bone tissue, osteoblasts and other types of sarcoma [36–38]. Transcript levels of the regulatory gene, *c-MYC*, are controlled by miRNAs at the 14q32 locus, and reinstating functional levels of these 14q32 miRNAs decreases c-MYC activity and induces apoptosis in Saos2 cells [36]. Consistent with findings in human OS, cross-species comparative analysis found decreased expression of miR-134 and miR-544 (orthologous to the human 14q32 miRNA cluster) in canine OS tumors compared to reactive canine osteoblasts [37]. Furthermore, reduced expression of 14q32 miRNAs in human OS tumors and orthologous miR-134 and miR-544 in canine OS is associated with shorter survival, suggesting that dysregulation of the 14q32 miRNA cluster may represent a conserved mechanism contributing to the aggressive biological behavior of OS in both species.

Given that canine OS is often used as a spontaneous large animal model of the human disease to test novel therapeutic approaches that may affect the course of microscopic metastasis, a detailed understanding of the shared molecular mechanisms would be ideal to more accurately inform future clinical studies. As such, the purpose of this study was to compare the miRNA expression profiles in primary OS tumor samples and normal osteoblasts to identify key miRNAs that may be contributing to the biologic aggressiveness of canine OS.

## Methods

### Cell lines, primary cell cultures, primary tumor samples

Canine OS cell lines OSA8 and OSA16 [5] were maintained in RPMI-1640 (Gibco Life Technologies, Grand Island, NY, USA) supplemented with 10 % fetal bovine serum, non-essential amino acids, sodium pyruvate, penicillin, streptomycin, L-glutamine, and HEPES (4-(2-dydroxethyl)-1-piperazineethanesulfonic acid) at 37 °C, supplemented with 5 % CO_2_ (media supplements from Gibco). Normal canine osteoblasts (catalog no. Cn406-05) Cell Applications Inc, San Diego, CA, USA) were cultured in canine osteoblast medium (Cell Applications Inc, catalog no. Cn417-500).

Primary canine osteoblast cultures were generated from trabecular bone isolated from the femoral heads of dogs undergoing total hip arthroplasty or femoral head ostectomy at the Ohio State University Veterinary Medical Center (OSU-VMC) as previously described [39]. Briefly, femoral heads were washed in buffered saline and trabecular bone was curetted to remove bone chips. Bone chips were washed and digested in serum-free Dulbecco’s modified Eagle medium (DMEM)/F12K medium (Gibco) supplemented with 239 U/mL collagenase type XI (Sigma, St. Louis, MO, USA), 2 mM L-glutamine, 50 μg/mL pencillin-streptomycin and transferred to a spinner flask in a humidified incubator at 37 °C with 5 % CO2 for 3–4 h. Following digestion of cellular material, the bone fragments were washed with buffered saline and plated into T25 flasks in calcium-free DMEM/F12 medium supplemented with 10 % fetal bovine serum, 50 μg/mL ascorbate (Sigma), 50 μg/mL pencillin-streptomycin, and 2 mM L-glutamine with changes of medium every 3–4 days. Osteogenic induction of confluent monolayer cultures was accomplished using DMEM/F12 (Gibco) medium supplemented with 10 % fetal bovine serum, 0.1 μM dexamethasone (Sigma), 10 mM β-glycerophosphate (Sigma), 50 μg/mL ascorbate (Sigma), 50 μg/mL pencillin-streptomycin, and 2 mM L-glutamine for 21 d with medium changes every 3–4 days [40]. Control cultures were maintained without osteogenic supplements. Cultures were evaluated for alkaline phosphatase expression using the Leukocyte Alkaline Phosphatase Kit (Sigma) according to the manufacturer’s instructions. The protocol for generation of canine osteoblasts was approved by the OSU Institutional Animal Care and Use Committee (IACUC, protocol 2009A0184). Normal canine tissue collections were approved by the OSU IACUC (protocol 2010A0015). Fresh frozen canine OS tumor samples were obtained from dogs presenting to the OSU-VMC and from Dr. Jaime Modiano at the University of Minnesota (UMN) Veterinary Medical Center. Tumor sample collections were performed in accordance with established hospital protocols and approved by the respective IACUCs at both OSU and UMN. Clinical patient data, including age, sex, breed, histopathological diagnosis, and primary tumor location is detailed in Additional file 1: Table S1.

### RNA isolation, cDNA synthesis, RT-PCR and quantitative real-time PCR

RNA was extracted from normal fresh frozen canine tissues (brain cortex, bone, liver, lymph node, kidney, skeletal muscle, spleen, thyroid), primary canine osteoblast cultures, osteoblast cells, OS cell lines, and fresh frozen primary OS tumors using TRIzol reagent (Invitrogen, Carlsbad, CA, USA) according to the manufacturer’s instructions. To confirm bone marker expression in primary osteoblast cultures, cDNA was generated using 1 μg of total RNA using Superscript III (Invitrogen) and 1/20 of the resultant cDNA was used for each PCR reaction in a total volume of 25 μl. Primers designed and utilized for canine ALP, BMP2, OP, and GAPDH are listed in Table 1. Standard PCR was performed with all primer sets and amplicon length verified by agarose gel electropohoresis and visualization of products using the Alpha Imager system (Alpha Innotech Corp, San Leandro, CA, USA).

**Table 1:** Primer sequences

Primers	Primer sequences
Canine ALP 245F	5’-CAT ACA ACA CCA ACG CTC AGG-3’
Canine ALP 582R	5’-GAC GTT GTG CAT GAG CTG GTA GGC-3’
Canine OPN 130F	5’-GTA AGT CCA ATG AAA GCC ATG ACG-3’
Canine OPN 468R	5’-CAT TGA AGT CAT CTT CCA TAC TC-3’
Canine OC 001F	5’-CAG CCT TCG TGT CCA AG-3’
Canine OC 193R	5’-GCC ATA GAA GCG CTG GTA AG-3’
Canine BMP2 151F	5’-GAG TCC GAG TTG CGG CTG CTC AG-3’
Canine BMP2 475R	5’-GTT CCT GCA TCT GTT CCC G-3’
Canine GSN 387F	5’-CTG CCA TCT TCA CGG TGC AGC-3’
Canine GSN 549R	5’-CAC GAC TTC ATT GGG GAC CAC GTG C-3’
Canine TGFBI 1771F	5’-GACATGCTCACCATCAACGG-3’
Canine TGFBI 1919R	5’-GCTGTGGAAACATCAGACTCTGCAG-3’
K9 GAPDHF	5’-GTCCATGCCATCACTGCCACCCAG-3’
K9 GAPDHR	5’-CTGATACATTGGGGGTGGGGACAC-3’
GAPDHF	5’-ACC ACA GTT CCA TGC CAT CAC-3’
GAPDHR	5’-TCC ACC ACC CTG TTG CTG TA-3’
18S V2F	5’-AAA TCC TTT AAC GAG GAT CCA TT-3’
18S V2R	5’-AAT ATA CGC TAT TGG AGC TGG A-3’

Real-time PCR was performed using the Applied Biosystems StepOne Plus Detection System (Applied Biosystems, Foster City, CA, USA). Human Taqman miRNA assays (Applied Biosystems) were used according to manufacturer’s instructions to quantify mature miRNA levels in available canine cell lines and tissues (miR-1, miR-9, miR-10b, miR-29a, miR-122, miR-126, miR-199b, miR-200c, miR-451; all mature miRNAs share 100 % sequence homology between dogs and humans). MiRNA-specific primers were used to convert 50 ng total RNA to first-strand cDNA, followed by real-time PCR with TaqMan probes. All samples were normalized to U6 snRNA. To validate changes in mRNA expression for selected genes affected by miR-9 expression, total RNA was collected and cDNA was generated as described above. Canine GSN and TGFBI mRNA was detected using Fast SYBR green PCR master mix (Applied Biosystems) according to the manufacturer’s protocol and primer sets are detailed in Table 1. Normalization was performed relative to 18S rRNA. All reactions were performed in triplicate and included no-template controls for each gene. Relative gene expression for all real-time PCR data was calculated using the comparative threshold cycle method [41]. Experiments were repeated 3 times using samples in triplicate.

### Quantitative real-time PCR analysis of formalin-fixed paraffin-embedded canine primary osteosarcoma tumor samples

Formalin-fixed paraffin-embedded (FFPE) primary canine OS tissues were obtained from the OSU-VMC Biospecimen Repository. H&E stained sections from a single random block from each patient were reviewed by a pathologist (OHI) to define and mark representative OS tumor regions. Using the marked H&E stained glass slide as a map, the corresponding areas of the unstained FFPE tissue block were identified and 15 targeted core samples of each cancerous tissue region were obtained. Tumor cores were then processed and RNA was isolated using the RecoverAll™ Total Nucleic Acid Isolation Kit for FFPE (Applied Biosystems) according to manufacturer’s recommendations. To quantify miR-9 expression, cDNA was generated and real-time PCR was performed using Human Taqman miRNA assays (Applied Biosystems) as described above.

### MiRNA expression profiling

MiRNA expression profiling of normal canine tissues (brain cortex, liver, lymph node, kidney, skeletal muscle, spleen, thyroid), 72 fresh primary OS tumors, 2 primary osteoblast cultures, and canine osteoblast cells (Cell Applications) was performed at the OSU Comprehensive Cancer Center Genomics Shared Resource using the multiplexed nanoString nCounter miRNA system (nanoString Technologies, Seattle, WA, USA) according to manufacturer’s protocol [42]. Total RNA (100 ng) was used as input material. Small RNA samples were prepared by ligating a specific DNA tag onto the 3’ end of each mature miRNA according to manufacturer’s instruction (nanoString Technologies). These tags normalized the melting temperatures of the miRNAs and provided identification for each miRNA species in the sample. Excess tags were then removed and the resulting material was hybridized with an nCounter Human (V2) miRNA Expression Assay CodeSet containing a panel of miRNA:tag-specific nCounter capture and barcoded reporter probes. Hybridization reactions were incubated at 65 °C overnight. Hybridized probes were purified and immobilized on a streptavidin-coated cartridge using the nCounter Prep Station (nanoString Technologies). nCounter Digital Analyzer was used to count individual fluorescent barcodes and quantify target RNA molecules present in each sample. For each assay, a high-density scan (600 fields of view) was performed.

### NanoString data analysis

Abundances of miRNAs were quantified using the nanoString nCounter gene-expression system [42]. Boxplot analysis did not detect obvious batch effect or poor sample integrity; therefore, all data were used for analysis. Raw data was normalized using internal positive control probes included in each assay and then a filtering step was applied. Internal negative control probes were used to determine a background threshold (2 standard deviations above the mean negative control probe count value) and if more than 90 % of the samples had miRNA expression lower than the background threshold cutoff value, those miRNAs were filtered out. After data filtering, a total of 519 miRNAs were used for analysis. Filtered data was quantile normalized and linear regressions were used to compare miRNA expression between tumor samples and normal osteoblast samples. A *p*-value of 1/519 = 0.0019 was used as a cutoff to claim for significance if controlling 1 false positive among the 519 tested miRNAs. Differential miRNA expression was determined by one-way analysis of variance (ANOVA) and *p*-values of <0.0019 were considered statistically significant.

### miR-9, anti-miR-9, and shGSN lentivirus infection

Lentiviral constructs obtained from Systems Biosciences (Mountain View, CA, USA) were packaged using the pPACKH1 Lentivector Packaging KIT (catalog no. LV500A-1) according to manufacturer’s instructions. Canine osteoblast cells (Cell Applications Inc.) and OSA16 cells (5 × 10^5^) were transduced with negative control empty lentivirus (catalog no. CD511B-1) or pre-miR-9-3 lentivirus (catalog no. PMIRH9-3PA-1). For reciprocal knock-down experiments, canine OSA8 cells (5 × 10^5^) were transduced with pGreenPuro Scramble Hairpin Control lentivirus (catalog no. MZIP000-PA-1) or miRZip-9 anti-miR-9 lentivirus (catalog no. MZIP9-PA-1). Briefly, 5 × 10^5^ cells were plated and left overnight in 10 % serum-containing medium. The following day, the medium was changed to Stemline (Gibco) with transfection agent TransDux (Systems Biosciences) and either empty control or pre-miR-9-3 virus (osteoblasts) or miRZip-9 or negative control scrambled virus (OSA8) was added to cells according to manufacturer’s protocol. FACS-mediated cell sorting based on GFP expression was performed 72 h post-transduction and miR-9 expression was evaluated by real-time PCR (Applied Biosystems).

Stable knock down of gelsolin (GSN) was performed using short hairpin RNA (shRNA) lentiviral constructs (pLKO.1:Hygro-shScramble and pLKO.1:Hygro-shGSN) and high-titer lentiviral stocks were generated as described in the Addgene's pLKO.1 protocol. pLKO.1:hygro plasmid was a kind gift from Bob Weinberg (Addgene plasmid #24150). Briefly, 1 × 10^5^ OSA8 cells were plated and left overnight in 10 % serum-containing medium. The following day, the medium was replaced with serum-free medium and target cells were infected with transfection agent TransDux (Systems Biosciences) and either pLKO.1:Hygro-Scramble or pLKO.1:Hygro-shGSN virus or TransDux alone for 24 h. Cells were cultured for 7–10 days in 10 %-serum-containing medium supplemented with 75 ug/mL Hygromycin-B (Life Technologies) for plasmid selection. Knockdown of GSN was confirmed by quantitative real-time PCR and Western blotting Cells were collected and processed for Western blotting as described below to detect levels of GSN and efficiency of knock down. Sequences of template canine DNA were as follows: pLKO.1-shGSN.1 (5’-CCCGCTGTTCAAGCAGTTCTT-3’) and pLKO.1-shGSN.2 (5’-CTGCAGTATGACCTCCACTAC-3’).

### Matrigel invasion assay

To assess the effects of miR-9 and GSN on invasion, cell culture inserts (8-μm pore size; Falcon) were coated with 100 μL of diluted (1:10) Matrigel (BD Bioscience, San Jose, CA, USA) to form a thin continuous layer and allowed to solidify at 37 °C for 1 h. Canine osteoblasts, OSA8 and OSA16 cell lines (5 × 10^4^/mL) transduced with control lentivirus, pre-miR-9-3 lentivirus, miRZip-9 lentivirus, or shGSN lentivirus were prepared in serum-free medium and seeded into each insert (upper chamber) and medium containing 10 % fetal bovine serum was placed in the lower chamber. The cells were incubated for 24 h to permit invasion through the Matrigel layer. Cells remaining on the upper surface of the insert membrane were wiped away using a cotton swab, and cells that had migrated to the lower surface were stained with crystal violet and counted in ten independent 20× high powered fields for each Matrigel insert. Experiments were repeated 3 times using samples in triplicate.

### Wound healing assay

To evaluate the effects of miR-9 on cell migration, canine osteoblasts transduced with control lentivirus or pre-miR-9-3 lentivirus and OSA8 cells transduced with scrambled control lentivirus or miRZip-9 lentivirus were seeded in complete medium and grown until confluent in 6-well plates. A gap was introduced in the cells by scraping with a P200 pipette tip and cells were placed in fresh medium containing 10 % fetal bovine serum. After 20 h (OSA8) or 24 h (osteoblasts), migration across the gap was evaluated by digital photography. Each experiment was repeated 3 times.

### Cell proliferation

Canine osteoblasts and OSA16 cells (2.5 × 10^3^) transduced with control lentivirus or pre-miR-9-3 lentivirus were seeded in triplicate in 96-well plates; non-transduced cells served as negative controls. After 24, 48, or 72 h of culture, media was removed and plates were frozen at −80 °C overnight before processing with the CyQUANT® Cell Proliferation Assay KIT (Molecular Probes, Eugene, OR, USA) according to the manufacturer’s instructions. Fluorescence was measured using a SpectraMax microplate reader (Molecular Devices, Sunnyvale, CA, USA). Cell proliferation was calculated as a percentage of non-transduced control cells. Each experiment was repeated 3 times.

### Detection of Apoptosis/Caspase 3/7 activity

Induction of apoptosis was assessed using the Senso-Lyte® Homogeneous AMC Caspase- 3/7 Assay KIT (Anaspec Inc., San Jose, CA, USA) as previously described [43]. Canine osteoblasts and OSA16 cells (2.5 × 10^3^) transduced with either empty lentivirus or pre-miR-9-3 lentivirus were plated in triplicate in 96-well plates for 24 and 48 h prior to analysis. Fluorescence was measured on a SpectraMax microplate reader (Molecular Devices) and caspase 3/7 activity was reported after subtraction of background fluorescence elicited by medium alone. Each experiment was repeated 3 times.

### 2D-DIGE and protein identification by LC-MS/MS

Protein lysates prepared from canine osteoblast cells transduced with either empty lentivirus (*n* = 4) or pre-miR-9-3 lentivirus (*n* = 4) were purified using the 2-D Clean-Up Kit (GE Healthcare, Uppsala, Sweden). Samples were suspended in 100 μL of lysis buffer (30 M Tris pH 8.5, 7 M Urea, 2 M Thiourea, 4 % CHAPS) and quantitated by Bradford assay. Two-dimensional difference gel electrophoresis (2D-DIGE) was performed as previously described [44]. Briefly, internal control samples were prepared by mixing a portion of all individual samples. Pooled internal control standards (50 μg) were labeled with Cy2 dye and individual samples (50 μg) were labeled with the appropriate Cy3 or Cy5 dye (GE Healthcare) according to the manufacturer's instructions, mixed and separated by two-dimensional difference gel electrophoresis. The first dimension separation was achieved using IPG isoelectric focusing strips (24 cm length, pH 3–10; GE Healthcare). The second dimension separation was achieved by SDS-PAGE on large-format gels (20x24cm) using a Dalt 12 electrophoresis system (GE Healthcare). Gels were scanned using a Typhoon 9400 variable mode scanner (GE Healthcare) at appropriate wavelengths. Gel images were analyzed and relative protein abundance was performed using SameSpots software (TotalLabs). Background subtraction, quantification and normalization were automatically applied with low experimental variation. The student’s *t*-test was used to compare protein expression for each spot between empty vector and miR-9-transduced osteoblast samples and *p*-values of < 0.05 were considered significant.

Protein spots of interest were located and excised from separate preparative gels using the Ettan Spot Handling Workstation (GE Healtchare) according to manufacturer’s instructions. Gel pieces containing protein spots were cored, digested with trypsin (Promega, Madison, WI, USA), and subjected to capillary-liquid chromatography-nanospray tandem mass spectrometry (Nano-LC/MS/MS) using a Thermo Finnigan LTQ mass spectrometer equipped with a nanospray ion source. The LC system used was an UltiMate™ Plus from LC-Packings A Dionex Co (Sunnyvale, CA, USA) with a Famos autosampler and Switchos column switcher. Mascot Daemon software (version 2.2; Matrix Science, Boston, MA, USA) was used to search for the mass of the peptide ion against other mammalian proteins in the NCBI database (1,391,110 sequences). Protein identifications were checked manually and proteins with a Mascot score of 100 or higher with a minimum of two unique peptides from one protein having a *-b* or *-y* ion sequence tag of five residues or better were accepted.

### RNA Sequencing

Total RNA was extracted from canine osteoblast cells transduced with either empty lentivirus (*n* = 3) or pre-miR-9-3 lentivirus (*n* = 3) using the TRIzol method and RNA sequencing was performed at the OSU Comprehensive Cancer Center Genomics Shared Resource. Briefly, total RNA was treated by Ribo-Zero Gold Subtraction reagents from TruSeq stranded total RNA (Illumina; RS-122-2201) to remove cytoplasmic and mitochondrial rRNAs and for the subsequent construction of the RNA-seq library according to the manufacturer’s instructions. Stranded total transcriptome libraries were quantified and qualified by Qubit and RIN analysis, respectively. Sequencing was performed on an Illumina HiSEq. 2500 instrument at a depth of ∼ 40 million paired-end, 100 bp long, strand-specific reads per sample. AdapterRemover was used to trim adapter sequences and the remaining reads were aligned using STAR to the canFam3 genome. Quality control was done using RNA-SeQC, in order to check for samples needing additional sequencing reads. Differential expression was determined using two different pipelines. First, gene expression counts were generated by HTSeq-Count and fed into DESeq2, which compared several sample groups using a likelihood-ratio test (LRT). Second, expression of individual transcripts was quantified by cuffquant with comparisons between pairs of conditions performed by cuffdiff.

Statistical analysis relative to mRNA expression data was performed using CuffDiff Software. Differential gene expression was determined by student’s *t*-test and *p*-values of <0.05 were considered statistically significant. Prediction of miR-9 binding to the 3’-UTR of genes down-regulated by miR-9 was performed with computer-aided algorithms obtained from TargetScan (http://www.targetscan.org), PicTar (http://pictar.mdc-berlin.de), miRanda (http://www.microrna.org), and miRWalk (http://www.umm.uni-heidelberg.de/apps/zmf/mirwalk).

### Immunoblotting

Protein lysates from canine osteoblasts transduced with control or pre-miR-9-3 lentivirus and OSA8 cells stably expressing scramble or miRZip-9 lentiviral constructs were prepared and quantified, separated by SDS-PAGE, and western blotting was performed as previously described [43]. The membranes were incubated overnight with anti-gelsolin antibody (D9W8Y, catalog no. 12953, Cell Signaling Technology, Danvers, MA) then incubated with the appropriate horseradish peroxidase linked secondary antibodies, washed, and exposed to substrate (SuperSignal West Dura Extended Duration Substrate, Pierce, Rockford, IL). Blots were stripped, washed, and reprobed for β-actin (Santa Cruz Biotechnology, Santa Cruz, CA).

### Statistics

Whenever possible, experiments were performed in triplicate and repeated 3 times. Data were presented as mean plus or minus standard deviation. Real time PCR miRNA or gene expression data was first normalized to internal control (U6 snRNA and 18S, respectively) and the delta delta Ct method [41] was used to compare miRNA expression by one-way ANOVA. For analysis of invasion assay data, a linear mixed effects model was used to take account of the correlations among observations run in the same biological replicate. Group comparisons in the CyQUANT® proliferation assays, caspase 3/7 activity, and invasion assays were analyzed by ANOVA. Values of *p* < 0.05 were considered statistically significant.

## Results

### A unique miRNA expression signature is associated with primary canine OS

To characterize miRNA expression in canine OS and evaluate the role of miRNA dysregulation in OS pathogenesis, it was first necessary to validate a method to analyze differential expression. The Human (V2) miRNA Expression Assay CodeSet was used to profile a panel of seven normal canine tissues (brain cortex, liver, lymph node, kidney, skeletal muscle, spleen, thyroid; *N* = 3 per tissue). Because of the high degree of sequence conservation of miRNAs across humans and dogs, 168 miRNAs on the human panel have sequences identical to canine miRNAs (miRBase v.15). A high abundance of tissue-specific miRNAs (see Additional file 2: Figure S1) were detected and real-time PCR was used to validate this finding (see Additional file 3: Figure S2). These miRNA abundance data from normal canine tissues establish that the nanoString nCounter platform is a valid high-throughput methodology to study miRNA expression in canine samples.

**Fig. 1:**
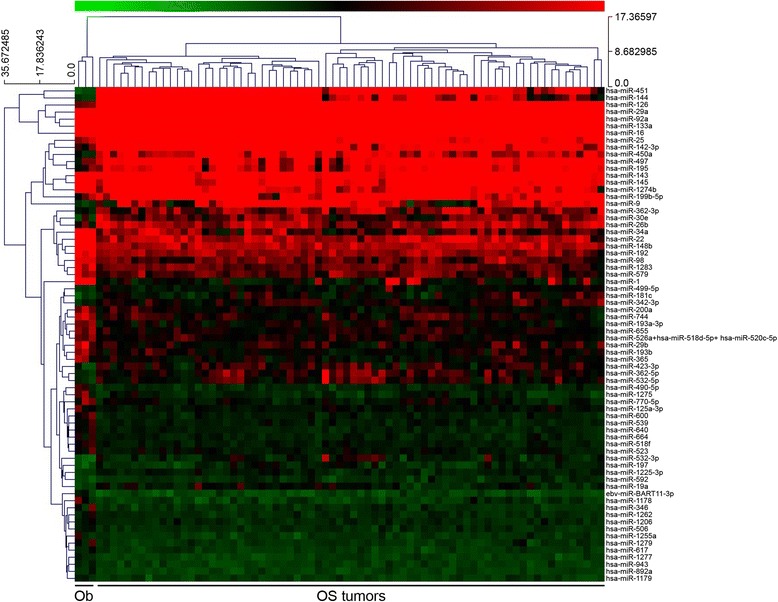
MiRNA expression signature associated with primary canine OS. MiRNA profiling was performed using the nanoString nCounter system to assess mature miRNA expression in fresh primary canine OS tumors (*n* = 72), primary canine osteoblast cultures (*n* = 2), and a canine osteoblast cell line (*n* = 1). Supervised hierarchical clustering was performed for 70 miRNAs differentially expressed in primary canine OS tumors (OS) compared to primary canine osteoblast cultures or cell lines (Ob) as determined by one-way ANOVA comparison test (*p* < 0.0019)

**Fig. 2:**
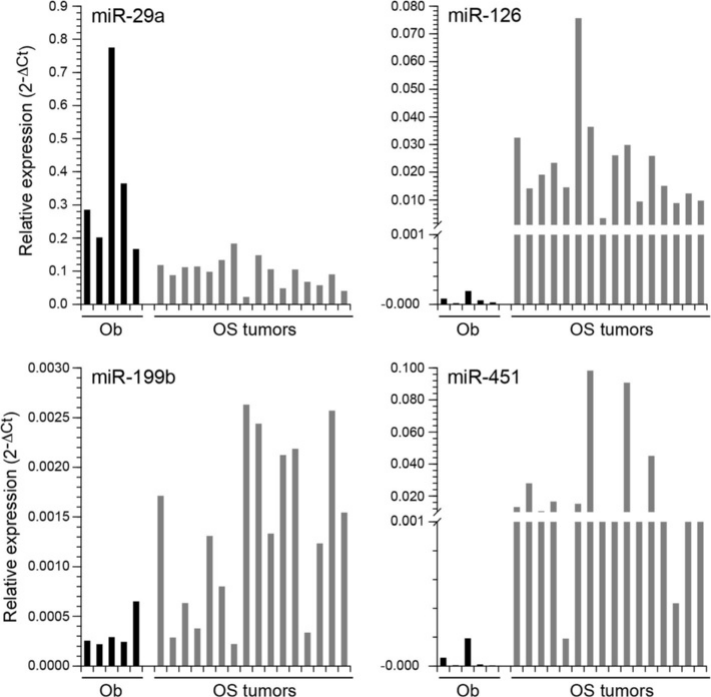
Expression of dysregulated miRNAs in canine OS. Real-time PCR was performed to independently validate changes in miRNA expression for 4 representative differentially expressed miRNAs (miR-29a, miR-126, miR-199b, miR-451) in a subset of primary canine osteoblasts cultures, osteoblast cell line (Ob, *n* = 5), and fresh primary canine OS tissues (OS, *n* = 16). Real time PCR confirmed overexpression of miR-126, miR-199b, and miR-451 and down-regulation of miR-29a expression in canine OS tumors as compared to normal canine osteoblasts (*p* ≤ 0.01). Three independent experiments were performed and all reactions were run in triplicate

To generate normal cells for comparison to osteosarcoma cells, primary osteoblast cultures were differentiated in vitro from canine mesenchymal stem cells. These cells were confirmed to express alkaline phosphatase and other bone-specific markers (Additional file 4: Figure S3). Global miRNA expression in fresh primary canine OS tumor samples (*N* = 72), primary osteoblast cultures (*N* = 2), and normal osteoblast cells (commercially available, *N* = 1) was evaluated using the nanoString nCounter platform. A distinct miRNA expression signature composed of 70 differentially expressed miRNAs was identified in primary canine OS tumor samples compared to canine osteoblast cells or primary osteoblast cultures (Fig. 1). We found 26 miRNAs that were significantly overexpressed in canine OS tumor samples compared to canine osteoblast cells or primary osteoblast cultures, while 44 miRNAs were downregulated in OS tumor tissues (Table 2). To validate these findings, real-time PCR confirmed differential expression for 4 of the 70 significant OS miRNAs among a random sampling of 16 fresh OS specimens and 5 control osteoblast cultures (Fig. 2). Specifically, we verified the overexpression of miR-126, miR-199b, miR-451 in OS samples relative to normal osteoblasts. In contrast, miR-29a showed significant down-regulation in primary canine OS tumor tissues compared to normal osteoblasts. Furthermore, several differentially expressed miRNAs identified in our canine OS tumor samples show similar alterations in human OS tumors (Table 2, bolded).

**Table 2:** MiRNA expression signature associated with canine osteosarcoma

miRNA	Fold-change expression OS vs Ob	*p*-value	miRNA	Fold-change expression OS vs Ob	*p*-value	miRNA	Fold-change expression OS vs Ob	*p*-value
Upregulated miRNAs			Downregulated miRNAs			Downregulated miRNAs		
**hsa-miR-126**	22.8	1.13E-13	hsa-miR-600	−2.2	1.75E-08	hsa-miR-1277	−1.6	7.94E-05
**hsa-miR-450a**	7.2	1.55E-08	hsa-miR-22	−4.1	3.37E-08	hsa-miR-98	−2.3	0.00011
**hsa-miR-451**	93.1	1.50E-07	**hsa-miR-34a**	−6.4	5.39E-08	hsa-miR-539	−1.7	0.00011
hsa-miR-30e	3.5	9.21E-06	hsa-miR-640	−1.8	2.27E-07	**hsa-miR-1**	−7.4	0.00011
hsa-miR-423-3p	3.0	1.93E-05	hsa-miR-655	−2.0	3.93E-07	hsa-miR-664	−1.7	0.00013
hsa-miR-532-3p	3.3	2.21E-05	hsa-miR-518f	−1.8	5.51E-07	hsa-miR-770-5p	−2.2	0.00015
**hsa-miR-92a**	2.6	2.89E-05	**hsa-miR-143**	−5.5	6.43E-07	**hsa-miR-29a**	−2.7	0.00020
**hsa-miR-25**	2.6	3.13E-05	**hsa-miR-145**	−5.4	6.79E-07	hsa-miR-365	−2.0	0.00020
**hsa-miR-142-3p**	6.8	6.52E-05	hsa-miR-1279	−2.1	1.48E-06	ebv-miR-BART11-3p	−1.9	0.00024
**hsa-miR-497**	4.5	7.69E-05	hsa-miR-200a	−2.3	4.35E-06	**hsa-miR-133a**	−2.1	0.00026
hsa-miR-499-5p	2.0	8.22E-05	hsa-miR-1283	−2.1	5.00E-06	hsa-miR-617	−1.6	0.00035
**hsa-miR-181c**	2.8	0.00013	hsa-miR-744	−2.7	6.23E-06	hsa-miR-1262	−1.6	0.00036
hsa-miR-342-3p	2.2	0.00013	hsa-miR-193b	−2.2	8.09E-06	hsa-miR-1274b	−5.2	0.00049
hsa-miR-362-5p	2.7	0.00015	hsa-miR-490-5p	−1.9	1.47E-05	hsa-miR-943	−1.6	0.00051
hsa-miR-144	20.8	0.00018	hsa-miR-1275	−2.5	2.16E-05	hsa-miR-1255a	−1.6	0.0012
**hsa-miR-195**	3.7	0.00026	hsa-miR-193a-3p	−2.2	2.18E-05	hsa-miR-579	−1.9	0.0013
hsa-miR-16	2.3	0.00037	hsa-miR-148b	−1.9	2.28E-05	hsa-miR-892a	−1.5	0.0013
hsa-miR-362-3p	3.0	0.00039	hsa-miR-1178	−2.4	3.22E-05	hsa-miR-125a-3p	−1.5	0.0014
hsa-miR-532-5p	2.5	0.00040	**hsa-miR-29b**	−2.7	3.59E-05	hsa-miR-523	−1.6	0.0015
hsa-miR-1225-3p	1.6	0.00050	hsa-miR-346	−1.9	4.18E-05	hsa-miR-1206	−1.5	0.0017
hsa-miR-26b	2.5	0.00052	**hsa-miR-192**	−1.9	4.28E-05	hsa-miR-506	−1.5	0.0018
**hsa-miR-199b-5p**	6.2	0.00054	hsa-miR-526a+	−1.8	3.10E-05	hsa-miR-1179	−1.5	0.0019
hsa-miR-197	2.2	0.00067	hsa-miR-518d-5p+					
**hsa-miR-9**	9.2	0.00087	hsa-miR-520c-5p					
hsa-miR-592	1.7	0.0014						
**hsa-miR-19a**	2.0	0.0018						

Bold indicates miRNAs similarly altered in human OS

### 1.3.2 miR-9 is up-regulated in canine OS tumor tissues and cell lines

**Fig. 3:**
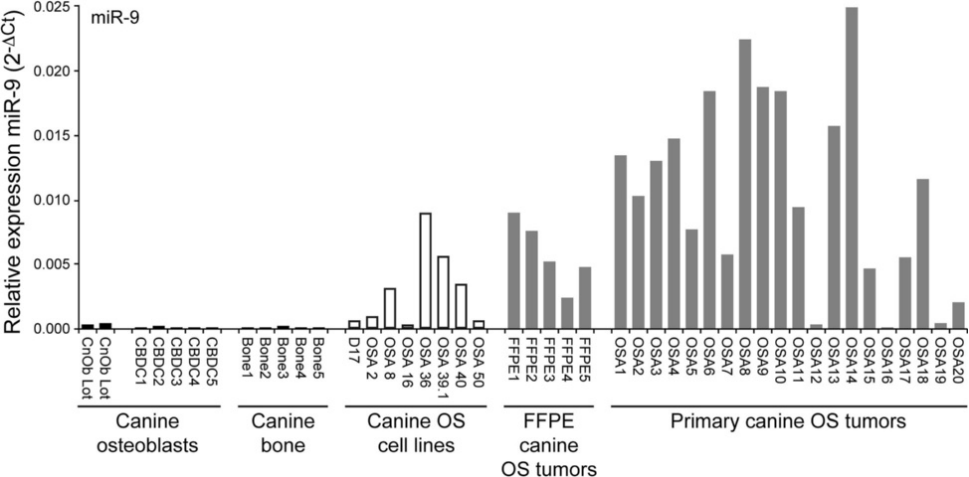
MiR-9 is highly expressed in primary canine OS tumors and canine OS cell lines. RNA was collected from primary canine osteoblast cultures (CBDC, *n* = 5), normal canine osteoblast cell lines (Ob, *n* = 2, corresponding to 2 different commercially available lots), normal canine bone (*n* = 5), canine OS cell lines (*n* = 8), FFPE canine OS tumor cores (*n* = 5), and fresh primary canine OS tumors (*n* = 20) and real-time PCR was performed to evaluate miR-9 expression. Real time PCR confirmed overexpression of miR-9 in canine OS tumors and OS cell lines as compared to normal canine osteoblasts (*p* < 0.001). Three independent experiments were performed and all reactions were performed in triplicate

Of the miRNAs found to be dysregulated in canine OS, miR-9 expression levels were significantly higher in primary canine tumor samples as compared to normal canine osteoblasts. This finding was independently validated by real-time PCR for miR-9 in fresh primary canine OS tumors, canine OS cell lines, normal canine bone, osteoblast cell lines, and primary osteoblast cultures (Fig. 3), demonstrating significantly higher levels of miR-9 expression in the tumors and OS cell lines relative to normal bone or osteoblasts. Primary osteosarcoma tumor specimens exhibit significant cellular heterogeneity; it was therefore possible that expression levels of miR-9 in tumor samples were influenced by the proportion of tumor cells to stroma/inflammatory cells. To assess the contribution of tumor microenvironment on miR-9 expression in primary canine OS tissues, we identified homogenous OS tumor cells regions in FFPE primary OS tumor specimens and isolated RNA from targeted tumor core samples. We found that miR-9 expression was markedly increased in OS tumor cells as compared to normal canine bone, normal canine osteoblasts and primary osteoblast cultures (Fig. 3). These findings demonstrate that the observed overexpression of miR-9 in canine OS tumor samples is not secondary to non-neoplastic cells infiltrating the tumor microenvironment, but derived directly from the malignant osteoblasts. These data are concordant with published data demonstrating overexpression of miR-9 in human OS [45].

### Overexpression of pre-miR-9 does not alter cellular proliferation or caspase-3,7 dependent apoptosis in normal canine osteoblasts or the OSA16 cell line

**Fig. 4:**
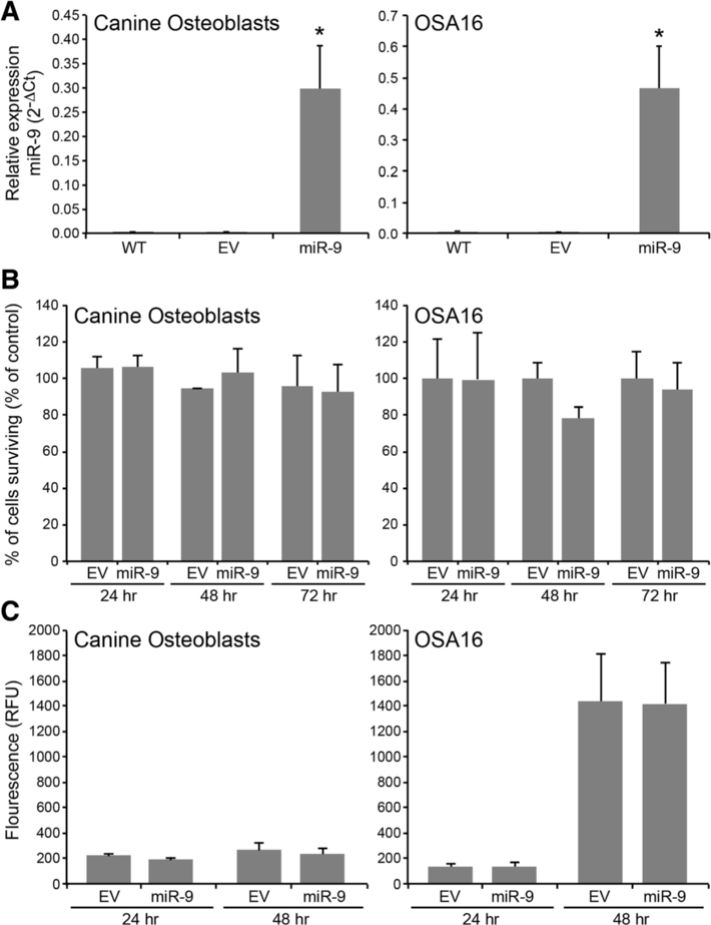
Overexpression of miR-9 has no effect on cell proliferation or apoptosis of normal osteoblasts or OS cell lines. **a** Normal canine osteoblasts and OSA16 cells transduced with pre-miR-9-3 lentivirus or empty vector control were sorted to greater than 95 % purity based on GFP expression. MiR-9 levels were assessed by real-time PCR in wild-type, empty vector, and miR-9 expressing cells (Bars: SD. Statistical analysis: one-way ANOVA, *p < 0.01). Three independent experiments were performed and all reactions were performed in triplicate. **b** Canine osteoblasts or OSA16 cells were transduced with either empty vector or pre-miR-9-3 lentivirus vector and cell proliferation was analyzed at 24, 48, and 72 h using the CyQUANT method. Non-transduced osteoblasts or OSA16 cells served as non-treated controls. Three independent experiments were performed and all samples were seeded in triplicate wells. Values are reported as percentage of untransduced control cells. **c** Canine osteoblasts or OSA16 cells transduced with either empty vector or pre-miR-9-3 lentivirus were assessed for apoptosis at 24 and 48 h by measuring active caspase-3/7 using the SensoLyte® Homogeneous AMC Caspase-3/7 Assay kit. Relative fluorescence units are reported after subtraction of fluorescence levels of wells with medium only

To assess the biological consequences of miR-9 expression in normal osteoblasts or malignant OS cell lines, canine osteoblasts and the OSA16 cell line that exhibits low endogenous expression of miR-9 were transduced with a pre-miR-9-3 lentiviral expression vector. Stably transduced GFP + cells were sorted and real-time PCR was used to confirm miR-9 overexpression (Fig. 4a). To determine the impact of miR-9 expression on normal or malignant osteoblast cell proliferation and apoptosis, canine osteoblasts and the OSA16 cell line expressing control or pre-miR-9-3 lentiviral constructs were cultured for 24, 48, and 72 h and cell proliferation and caspase-3,7 activity was assessed. Overexpression of miR-9 had no observed effects on cell proliferation or apoptosis in either normal osteoblast cells or malignant OS cell lines (Fig. 4b, c).

### miR-9 expression enhances invasion and migration in normal osteoblasts and the OSA16 cell line

**Fig. 5:**
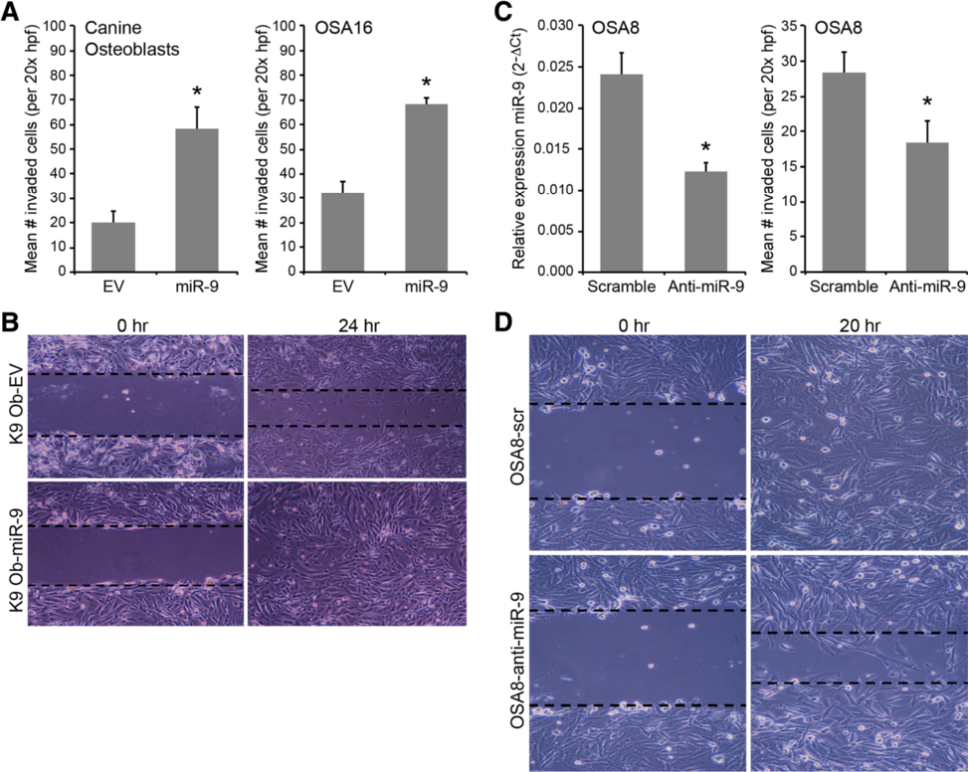
MiR-9 enhances invasion and migration in normal canine osteoblasts and OS cell lines. **a** The invasive capacity of normal canine osteoblasts or OSA16 cells transduced with either empty vector or pre-miR-9-3 lentivirus was evaluated using standard Matrigel invasion assays. Cells (5 × 10^4^) were plated in serum free medium and transferred onto cell culture inserts coated with Matrigel® for 24 h. After incubation, cells remaining on the upper surface of the insert membrane were wiped away using a cotton swab, and cells that had migrated to the lower surface were stained with crystal violet and counted in ten independent 20x hpf for each sample. Three independent experiments were performed and all assays were performed in triplicate wells (Bars: SD. Statistical analysis: one-way ANOVA, *p < 0.001). **b** Cell migration was assessed in canine osteoblasts transduced with either empty vector or pre-miR-9-3 lentivirus using standard wound-healing assays. Cells were seeded in complete medium and grown until confluent in 6-well plates. A gap was created using a P200 pipette tip, and medium was replaced with serum-free medium. After 24 h, cell migration was evaluated by digital photography. **c** Canine OSA8 cells were transduced with miRZip-9 (anti-miR-9) or scramble vector and miR-9 levels were assessed by real-time PCR to confirm transduction efficiency (*p < 0.0009). Cell invasion was assessed in canine OSA8 cells transduced with scramble or miRZip-9 (anti-miR-9) lentivirus using standard Matrigel invasion assays as described above. Three independent experiments were performed and all assays were performed in triplicate wells (*p < 0.0002). **d** Cell migration was assessed in OSA8 cells transduced with miRZip-9 (anti-miR-9) or scramble vector using standard wound-healing assays as described above. After 20 h, cell migration was evaluated by digital photography

To investigate the effects of enforced miR-9 expression on invasive capacity, a standard Matrigel Invasion assay was performed to evaluate cell invasion. As shown in Fig. 5a, overexpression of miR-9 in normal osteoblasts or malignant OSA16 cells significantly enhanced their invasion after 24 h of culture compared to cells expressing empty vector. Cell migration activity was assessed in normal osteoblasts overexpressing miR-9 using the wound-healing assay (scratch test). Fig. 5b demonstrates that miR-9 enhanced cell motility and scattering following gap formation in normal osteoblasts compared to osteoblasts expressing control vector (Fig. 5b). Collectively, these findings demonstrate that miR-9 promotes an invasive phenotype in normal and malignant canine osteoblasts.

### Anti-miR-9 expression decreases cell invasion and migration in OSA8 cells

To determine whether inhibition of miR-9 would impair cell migration and invasion, the canine OSA8 cell line that expresses high basal levels of miR-9 was transduced with miRZip-9 (anti-miR-9) or control lentivirus. Transduced cells were sorted based on GFP expression and miR-9 expression was assessed by quantitative PCR to confirm mature miR-9 knockdown (Fig. 5c). In concordance with our findings in normal canine osteoblasts and OSA16 cells overexpressing miR-9, inhibition of miR-9 in OSA8 cells significantly decreased cell invasion and migration compared to control cells (Fig. 5c, d), providing further support for the role of miR-9 in OS invasion.

### 2D-DIGE electrophoresis and RNA sequencing identifies miR-9-induced alterations to the proteome and transcriptome of canine osteoblasts

**Fig. 6:**
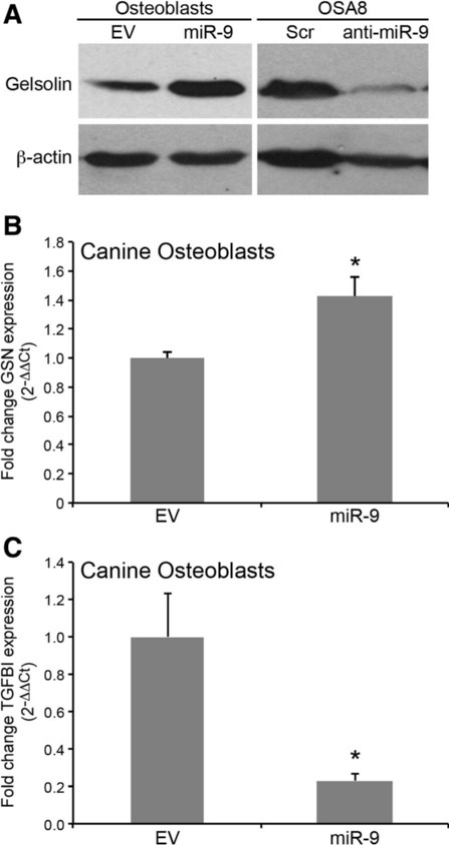
MiR-9 alters gelsolin expression in normal canine osteoblasts and OS cell lines. **a** Protein lysates were generated from normal canine osteoblasts stably transduced with either empty vector (EV) or pre-miR-9-3 (miR-9) lentivirus and canine OSA8 cells stably transduced with miRZip-9 (anti-miR-9) or scramble vector control. Protein lysates were separated via SDS-PAGE and western blotting for gelsolin and β-actin was performed. **b** Canine osteoblasts expressing pre-miR-9-3 lentivirus or empty vector control were collected and real-time PCR for gelsolin and (**c**) TGFBI was performed (Bars: SD. Statistical analysis: one-way ANOVA, *p < 0.01). Three independent experiments were performed using cells from 3 separate transduction experiments and all reactions were performed in triplicate

To gain further mechanistic insight into miR-9-dependent cell signaling events that may promote the invasive phenotype of osteoblasts, we analyzed the proteomic and gene expression profiles of canine osteoblasts expressing control or miR-9 lentiviral constructs. Two-dimensional difference-in-gel electrophoresis (2D-DIGE) identified 10 protein spots that were differentially expressed in osteoblasts overexpressing miR-9 compared to cells expressing empty control vector (Additional file 5: Figure S4). Determination of the proteins located at these spots was undertaken using in-gel trypsin digestion followed by tandem mass spectrometry (Table 3). Four of the protein spots were unable to be definitively identified, and 2 of the proteins found to be significantly down-regulated following miR-9 overexpression did not have putative miR-9 binding sites within their 3’-UTR, implying that miR-9 may indirectly regulate their expression. Interestingly, miR-9 induced up-regulation of several proteins involved in actin dynamics and cytoskeletal remodeling, including gelsolin and cofilin-1. To independently validate these changes in protein expression, western blotting was performed for gelsolin, an actin binding protein implicated in neoplastic transformation and metastasis [46, 47]. Consistent with our 2D-DIGE results, gelsolin (GSN) was up-regulated in miR-9 expressing osteoblasts (Fig. 6a). Concordant with these results, GSN protein expression was substantially reduced following down-regulation of miR-9 in OSA8 cells transduced with anti-miR-9 vector as compared to cells expressing control vector (Fig. 6a). Furthermore, real time PCR demonstrated an increase in GSN mRNA expression in osteoblasts overexpressing miR-9 compared to empty vector controls, which was further validated with RNA sequencing (0.4-fold increase, *p* = 0.05) (Fig. 6b).

**Table 3:** Proteins identified by two-dimensional electrophoresis and capillary-liquid chromatography-tandem mass spectrometry

Spot #	*t*-test	Av Δ	Acc #	Description	pI	Mass	S^a^	M^b^
291	0.038	−1.4	gi|73997540	PREDICTED: ELKS/Rab6-interacting/CAST family member 1 isoformX1 [Canis lupus familiaris]	5.69	128164	147	2
919	0.007	−1.1	gi|73980394	PREDICTED: protein disulfide-isomerase A6 [Canis lupus familiaris]	4.97	48667	906	11
961	0.021	−1.3	No ID					
1080	0.034	1.1	No ID					
1384	0.033	1.2	No ID					
1609	0.027	1.4	gi|5031635	cofilin-1 [Canis lupus familiaris]	8.52	19715	343	3
1617	0.03	1.2	No ID					
1872	0.029	1.2	gi|478533920	PREDICTED: 60S acidic ribosomal protein P2 [Ceratotherium simum simum]	4.44	11772	364	4
1925	0.052	1.2	gi|350582724	PREDICTED: 14-3-3 protein theta isoform 2 [Sus scrofa]	4.67	28075	983	12
1935	0.008	1.2	gi|545518174	PREDICTED: gelsolin [Canis lupus familiaris]	8.49	95129	609	8

^a^The protein score is derived from Mascot and provides an indication of how well the peptides matched the indicated protein sequence. The actual score is calculated by the following equation: protein score = −10*Log(P), where P is the probability that the protein match is a random event. Scores above 100 indicate that *p* < 0.05

^b^The protein match score indicates the number of unique peptides that matched the sequence of the identified protein. Two unique peptide matches to a protein sequence confirms the identity of a prot

We compared the gene expression profile of osteoblasts possessing enforced miR-9 expression to that of cells expressing empty control vector and observed significant differences in transcript expression. RNA sequencing identified 55 transcripts that were significantly up-regulated (>2-fold) and 139 transcripts were significantly down-regulated in osteoblasts overexpressing miR-9 (Additional file 6: Table S2). Consensus binding sites for miR-9 were identified within the 3’-UTR of 37 genes that were significantly downregulated following miR-9 overexpression, suggesting that miR-9 may regulate the expression of these putative target genes (Additional file 6: Table S2, bolded). The transcripts identified as predicted targets of miR-9 are involved in a variety of cellular processes, including transcription (transcription factor 19, TCF19 and homeobox B6, HOXB6), RNA methyltransferases (NOL1/NOP2/Sun domain family, member 7, NSUN7), cytokinesis/microtubule assembly (protein regulator of cytokinesis 1, PRC1; kinesin family member 23, KIF23; stathmin 1, STMN1; and cancer susceptibility candidate 5, CASC5), endopeptidase activity (membrane metallo-endopeptidase, MME), and extracellular matrix organization (TGF-β-induced, TGFBI and collagen type IV, alpha 4–1 and −2, COL4A1, COL4A2). Interestingly, one of the most significantly down-regulated genes was TGFBI, an extracellular matrix protein and known mediator of osteoblast adhesion. TGFBI has several highly conserved predicted miR-9 binding sites within the 3’UTR indicating direct regulation of expression by miR-9. Concordant with our RNA sequencing results, real time PCR demonstrated downregulation of TGFBI transcript expression in canine osteoblasts overexpressing miR-9 (Fig. 6c).

### GSN shRNA decreases cell invasion and migration in canine OSA8 cells

**Fig. 7:**
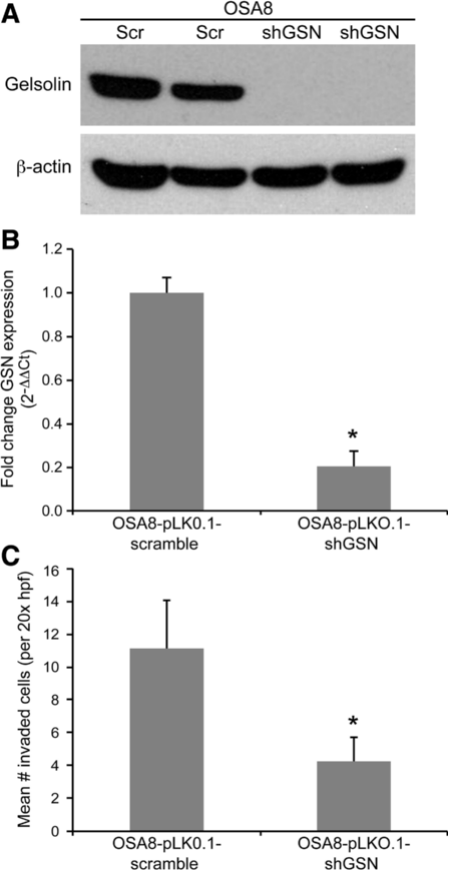
Gelsolin shRNA decreases cell invasion and migration in canine OSA8 cells. **a** Protein lysates were generated from canine OSA8 cells stably transduced with either pLK0.1-scramble-hygroB (Scr) control vector or pLK0.1-shGSN-hygroB (shGSN) lentivirus and positive clones were selected for with Hygromycin B. Protein lysates were separated via SDS-PAGE and western blotting for gelsolin (GSN) and β-actin was performed to confirm efficiency of GSN knockdown. **b** Canine OSA8 cells expressing pLK0.1-shGSN-hygroB or scramble control were collected and real-time PCR for gelsolin was performed (Bars: SD. Statistical analysis: one-way ANOVA, *p < 0.01). **c** The invasive capacity of OSA8 cells transduced with either pLK0.1-scramble-hygroB control vector or pLK0.1-shGSN-hygroB lentivirus was evaluated using standard Matrigel invasion assays. Cells (5 × 10^4^) were plated in serum free medium and transferred onto cell culture inserts coated with Matrigel® for 24 h. After incubation, cells remaining on the upper surface of the insert membrane were wiped away using a cotton swab, and cells that had migrated to the lower surface were stained with crystal violet and counted in ten independent 20x hpf for each sample. Three independent experiments were performed using cells from 3 separate transduction experiments and all reactions were performed in triplicate

Our previous findings support the notion that miR-9 promotes cell invasion and migration in canine osteoblasts, in part, through up-regulation of GSN. Given the role of gelsolin in the regulation of actin polymerization and cycling, we designed lentiviral-shRNA for canine GSN to determine the impact of GSN downregulation on cell invasion and migration in canine OSA8 cells. Expression of GSN was significantly reduced in OSA8 cells transduced with GSN shRNA as evidenced by Western blotting and quantitative real-time PCR (Fig. 7a, b). Furthermore, downregulation of GSN correlated with a significant decrease in cell invasion in canine OSA8 cells transduced with GSN shRNA as compared to those transduced with scrambled control shRNA (Fig. 7c) demonstrating a direct role for GSN in contributing to the invasive properties of osteosarcoma cells.

## Discussion

MiRNAs regulate numerous biological processes and their functions extend to both physiological and pathological conditions, including cell fate specification, cell death, development, and metabolism. Global dysregulation of miRNAs is a common feature of many human malignancies and miRNAs play an essential role in the development and progression of cancer by functioning as tumor suppressor genes or oncogenes. [11]. Similar to the case in human cancers, aberrant expression of miRNAs has been demonstrated in naturally occurring canine cancers indicating that miRNA dysregulation likely contributes to tumorigenesis in this species as well [28–31, 37, 48]. While the identification and function of altered miRNAs has been demonstrated in carcinomas and hematopoietic neoplasia, the influence of miRNAs in sarcomagenesis is incompletely understood. To date, the most detailed work has identified miRNA signatures associated with the pathogenesis and progression of human OS [20, 23, 25, 26]. The purpose of this study was to investigate the potential contribution of aberrant miRNA expression to the aggressive biological behavior of canine OS, a well-established spontaneous model of the human disease, and evaluate the functional consequences of altered miRNA expression in canine osteoblasts and OS cell lines.

We identified a unique miRNA signature associated with primary canine OS tumors as compared to normal canine osteoblasts. Significantly, several critical members of this signature in canine OS tumors have been reported as aberrantly expressed in human OS tumors [20–22, 49] or have known functions in human OS cell lines [50–55]. For example, miR-29a is frequently downregulated in human OS and regulates osteoblastic cell apoptosis by silencing Bcl-2 and Mcl-1 and inducing E2F1 and E2F3 expression [53]. The miR-34 family induces apoptosis and cell cycle arrest in a p53-dependent manner and is downregulated in primary OS tissue samples and OS cell lines. Enforced expression of miR-34a in OS cells was found to decrease expression levels of c-Met and several factors in the Wnt and notch signaling pathways in vitro and xenograft studies demonstrated that mice injected with stably transduced miR-34a OS cells had fewer metastases in vivo [55]. Lastly, overexpression of miR-199b-5p in human OS cell lines activates Notch signaling and promotes cell proliferation. Transfection with a miR-199b-5p inhibitor diminished cell invasiveness and decreased Notch signaling by reducing HES1 expression [52]. In concordance with these data, our findings indicate that these miRNAs are dysregulated in a similar manner in spontaneous canine OS. A functional approach to evaluate the consequences of each of these miRNAs in canine OS was outside the scope of the current study; however, together these findings support the idea that these miRNAs contribute to the development and/or progression of both canine and human OS.

Our data demonstrate that miR-9 expression is significantly upregulated in canine primary OS tumors compared to normal osteoblast cells or primary osteoblast cultures. Furthermore, we found that OS cells isolated from FFPE canine primary OS tumors express high levels of miR-9 compared to normal canine bone, canine osteoblasts and primary osteoblast cultures, suggesting that increased expression of miR-9 in OS tumors is primarily influenced by OS tumor cells and not stroma/inflammatory cells present in the tumor microenvironment. These findings are consistent with recent studies demonstrating that miR-9 expression is significantly increased in primary human OS tumors compared to paired non-cancerous bone tissues [45]. Furthermore, increased serum miR-9 concentrations in human OS patients and miR-9 expression in primary OS tumors strongly correlates with tumor size, clinical stage, distant metastasis, and poor clinical outcome [45, 56]. In concordance with data implicating miR-9 in promoting the aggressive biological behavior of osteoblasts, most of the malignant canine OS cell lines evaluated in this study expressed significantly higher levels of miR-9 compared to that observed in normal canine osteoblast cells and primary osteoblast cultures.

MiR-9 is known to confer multiple, often divergent, functions to various cell types including inhibition of human ovarian tumor cell growth [57] and stimulation of proliferation of gastric cancer cell lines [58]. Indeed, a common theme has emerged where miRNA effects are often cell type/tissue specific and may influence several aspects of cellular behavior. [11, 12]. Previous studies have identified a role for miR-9 in promoting the metastatic phenotype in human breast cancer cell lines through its ability to enhance cell invasion and enable otherwise non-metastatic breast tumor cells to form pulmonary micrometastases in mice [59]. Furthermore, miR-9 expression levels were shown to be significantly increased in distant metastases compared to corresponding primary tumors, suggesting that miR-9 is directly involved in the metastatic process [60, 61]. With respect to the role of miR-9 in spontaneous canine neoplasia, we have previously shown that overexpression of miR-9 is associated with aggressive, metastatic behavior in primary canine mast cell tumors and that enforced expression of miR-9 expression in normal and malignant mast cells enhances their invasive capacity and induces a pattern of gene expression that promotes cellular invasion [28]. In murine pre-osteoblasts and pluripotent stem cells, miR-9 expression is reduced following BMP2-induced osteoblastic differentiation, implying that miR-9 may be an important regulatory factor in osteoblastic differentiation [62, 63]. Other studies have shown that in human HOS and U2OS osteosarcoma cells express significantly higher levels of miR-9 compared to human mesenchymal stem cells or normal osteoblasts and demonstrate a functional role for miR-9 regulating osteosarcoma cell proliferation by targeting the GCIP tumor suppressor protein [64]. However, our data indicate that while enforced miR-9 expression in normal canine osteoblasts and the OSA16 cell line enhanced cellular invasion and migration, miR-9 had no impact on cellular proliferation or viability. Furthermore, transduction of the canine OSA8 cell line expressing high basal levels of miR-9 with an anti-miR-9 construct subsequently decreased invasion and migration, supporting the assertion that miR-9 promotes a metastatic phenotype in osteoblasts and OS cell lines.

To gain further mechanistic insight into miR-9-dependent cell signaling events that may promote the invasive phenotype of osteoblasts, we evaluated the proteomic and gene expression profiles of canine osteoblasts expressing high levels of miR-9. We did not identify predicted miR-9 targets among the proteins that were found to be down-regulated in our proteomic analysis. One possible explanation for this is that while the miRNA target prediction tools (TargetScan, miRanda, miRWalk, PicTar) used in this study identified miR-9 canonical binding sites in the 3’UTR, because miRNAs are known to target other non-canonical sites, coding regions or 5’ UTRs these would not be detected in our analysis. Interestingly, our proteomic analysis identified several proteins upregulated by miR-9, including gelsolin, an actin filament severing and capping protein implicated in promoting the metastatic phenotype [46, 65]. The importance of gelsolin in actin dynamics has been explored in gelsolin null (GSN-) dermal fibroblasts, which show a marked reduction in motility and ruffling activity in response to serum or EGF stimulation compared to wild-type fibroblasts [66]. In contrast, overexpression of gelsolin in NIH 3 T3 fibroblasts leads to a dose-dependent increase in translocation motility as assessed by tissue culture wound closure and filter transmigration assays [67]. Gelsolin-null (GSN-) mice demonstrate platelet shape changes causing prolonged bleeding times, delayed neutrophil migration, and blunted fibroblast responses, establishing the requirement of gelsolin for rapid motility in cell types involved in stress responses in vivo [68]. Importantly, elevated levels of gelsolin expression have been detected in human non-small cell lung [69], colorectal [70], and pancreatic carcinomas [71] and depletion of gelsolin using siRNAs in pancreatic, prostate, colorectal, and breast carcinoma cell lines caused a marked reduction in cell motility [72].

We confirmed significant upregulation of gelsolin transcript and protein in normal osteoblasts expressing miR-9 and found that knockdown of miR-9 in the OSA8 cell line resulted in decreased expression of gelsolin with a concomitant decrease in cell invasion and migration. Given the established role of gelsolin in the regulation of actin polymerization and cycling, our data indicate that miR-9 may enhance invasion in canine neoplastic osteoblasts, in part, through increased expression of this protein. To further investigate the effects of gelsolin on osteoblast motility, transduction of OSA8 cells with GSN shRNA downregulated GSN expression resulting in a subsequent decrease in cell invasion. Taken together, our findings support the idea that miR-9 induces a pattern of gene expression that promotes increased cell motility and invasiveness and suggest that miR-9-mediated up-regulation of gelsolin may in part play a role in mediating the biological consequences of miR-9. As miR-9 overexpression is associated with upregulation of gelsolin at the transcript and protein level, it is possible that miR-9 alters transcription factors responsible for repressing gelsolin expression. Alternatively, miR-9 may downregulate epigenetic factors that suppress gelsolin expression through DNA methylation or acetylation. Indeed, RNA sequencing of osteoblasts possessing enforced expression of miR-9 demonstrated that miR-9 modulated the expression of several genes, including downregulation of putative miR-9 targets whose transcripts contain 3’-UTR consensus binding sites for miR-9. Among these predicted targets, several transcription factors (HOX6B, TCF19) showed significant decreases in expression and putative HOXB binding sites were found in the promoter region of the canine gelsolin *(GSN)* gene, indicating a possible mechanism through which miR-9 induces upregulation of gelsolin.

Our data also show that miR-9 negatively regulates the expression of several other factors that may cooperatively enhance invasion and motility in normal osteoblasts. For example, miR-9 overexpression in normal osteoblasts downregulated expression of TGF-β-induced (TGFBI), an extracellular matrix protein and known mediator of osteoblast adhesion by virtue of its interactions with αvβ3 and αvβ5 integrin heterodimers [73]. TGFBI deficiency predisposes mice to spontaneous tumor development (lymphoma, lung adenocarcinoma) and TGFBI−/− mice have reduced body size, bone mass, bone size, and decreased periosteal bone formation, suggesting that TGFBI functions as a tumor suppressor and plays an important role in regulating bone homeostasis in vivo [74, 75]. A functional approach would be required to confirm direct targeting of putative gene targets by miR-9 and validate regulation of gene expression by miR-9. Furthermore, loss or gain of function studies evaluating components of the miR-9 regulatory circuit would further elucidate their contribution to osteoblast invasion and represents an ongoing area of investigation.

## Conclusions

Our data demonstrate that a unique miRNA expression signature is associated with spontaneously occurring canine OS. Furthermore, primary canine OS tumor specimens and OS cell lines express significantly higher levels of miR-9 compared to normal canine osteoblasts and primary osteoblast cultures. These results are concordant with data generated in human OS tumors, suggesting that dysregulation of miR-9 may be fundamental to the disease process in both species. Our data indicate that overexpression of miR-9 in normal osteoblasts and OS cell lines contributes to the aggressive biological behavior of OS as demonstrated by enhanced cellular invasiveness and motility and alteration in gene and protein expression profiles associated with cellular invasion, thereby promoting the metastatic phenotype. Furthermore, the actin filament-severing protein gelsolin was identified as a mediator of the miR-9 induced invasive phenotype in normal osteoblasts and OS cell lines, providing a potential mechanism for the relationship between miR-9 expression and metastasis. Future work to more thoroughly characterize how miR-9 expression imparts a metastatic phenotype in OS is ongoing with the ultimate goal of identifying novel targets for therapeutic intervention.

## Additional files

Additional file 1: Table S1. Clinical Patient Data. (DOCX 18 kb)
Additional file 2: Figure S1. MiRNA expression profiling of normal canine tissues using the nanoString nCounter platform. MicroRNA expression was evaluated in normal canine tissues (brain cortex, liver, lymph node, kidney, skeletal muscle, spleen, thyroid; *N* = 3 per tissue) using the Human (V2) miRNA Expression Assay CodeSet. Hierarchical clustering was performed for 110 miRNAs demonstrating unique tissue-specific expression profiles as determined by one-way ANOVA comparison test (*p* < 1e-06). (TIF 5836 kb)
Additional file 3: Figure S2. Validation of nanoString profiling data in normal canine tissues. Real-time PCR was performed to independently validate changes in tissue-specific miRNA expression in normal canine tissues identified by the nanoString nCounter system. Real-time PCR confirmed differential expression of 5 representative miRNAs (miR-1, miR-9, miR-10b, miR-122, miR-200c) in normal canine tissues (brain cortex, liver, lymph node, kidney, skeletal muscle, spleen, thyroid; *N* = 3 per tissue) (Bars: SD. Statistical analysis: one-way ANOVA, **p* < 0.05). Three independent experiments were performed and all reactions were run in triplicate. (PNG 163 kb)
Additional file 4: Figure S3. Expression of bone markers in primary canine osteoblast cultures. (A) RNA was collected from five canine osteoblast (Ob) cultures established from canine patients, normal canine osteoblast cells (K9 Ob), and a canine OSA cell line (OSA8) and RT-PCR was performed for alkaline phosphatase (ALP), bone morphogenic protein-2 (BMP2), osteopontin (OP), and GAPDH. NTC = non-template control. (B) Primary canine osteoblast cultures were evaluated for ALP expression using immunocytochemistry (Sigma). Upper left panel: horse neutrophils (positive control), Upper right panel: stem cells cultured in non-differentiating conditions (negative control), Lower two panels: expression of ALP in differentiated primary canine osteoblast cultures. (TIF 9327 kb)
Additional file 5: Figure S4. 2-dimensional difference-in-gel electrophoresis (A) Representative gel image showing Cy3 (Ob-miR-9) and Cy5-labeled (Ob-EV) proteins that were isoelectric focused on pH strips (3-10), separated by size using SDS-PAGE, and visualized using a Typhoon 9400 variable mode imager. A merged image of the Cy3 and Cy5-labeled proteins is shown. Proteins with greater abundance in the miR-9-transfected sample appear green and proteins with greater abundance in the EV-transfected samples appear red. Proteins that did not change relative abundance between the two samples appear yellow. (B) Master gel image of spots exhibiting statistically significantly protein expression changes. Core protein spots of interest were excised from preparative gels stained with Lava purple general protein stain, digested with trypsin, and subject to capillary-liquid chromatography-tandem mass spectrometry analysis. The spots that were cored for subsequent protein identification are outlined and annotated (the number for each spot is the master spot number and corresponds to the Spot # listed in Table 3). (TIF 8721 kb)
Additional file 6: Table S2. Altered gene transcripts in canine osteoblasts overexpressing miR-9 (DOCX 18 kb)

## Abbreviations

2D-DIGE: Two-dimensional difference-in-gel electrophoresis
3’-UTR: 3’-untranslated region
ALP: alkaline phosphatase
BMP2: bone morphogenic protein-2
CASC5: cancer susceptibility candidate 5
COL4A1: collagen type IV, alpha 4–1
COL4A2: collagen type IV, alpha 4–2
FFPE: Formalin-fixed paraffin-embedded
GSN: gelsolin
HOXB6: homeobox B6
KIF23: kinesin family member 23
miRNA: microRNA
MME: membrane metallo-endopeptidase
NSUN7: NOL1/NOP2/Sun domain family, member 7
OP: osteopontin
OS: osteosarcoma
PRC1: protein regulator of cytokinesis 1
STMN1: stathmin 1
TCF19: transcription factor 19
TGFBI: TGF-β-induced
