# Supplementary material for: MiR-9 is overexpressed in spontaneous canine osteosarcoma and promotes a metastatic phenotype including invasion and migration in osteoblasts and osteosarcoma cell lines
Source: BMC Cancer. 2016 Oct 10;16:784. doi: 10.1186/s12885-016-2837-5 (PMC5057229; doi:10.1186/s12885-016-2837-5)
Supplement: Additional file 1: Table S1. — Clinical Patient Data. (DOCX 18 kb) [file 12885_2016_2837_MOESM1_ESM.docx]

**Table S1 Clinical Patient Data**

| **Breed** | **Sex/Neuter Status** | **Age (Yr)** | **Primary Tumor Location** | **Histopathologic Diagnosis** |
| --- | --- | --- | --- | --- |
| Greyhound | MC | 7 | Distal femur | Osteoblastic osteosarcoma |
| Greyhound | MC | 6 | Left distal radius | Osteogenic osteosarcoma |
| Greyhound | FS | 5 | Proximal humerus | Well-differentiated osteosarcoma |
| Greyhound | MC | 7 | Left distal radius | Osteoblastic osteosarcoma |
| Golden Retriever | F | 1.5 | Right distal radius | Osteoblastic osteosarcoma |
| Greyhound | MC | 6 | Left proximal humerus | Osteoblastic osteosarcoma |
| Great Pyrenes | MC | 8 | Right distal radius | Osteoblastic osteosarcoma |
| Greyhound | MC | 11 | Left distal tibia | Osteosarcoma |
| Mix breed | MC | 10 | Right proximal humerus | Well-differentiated osteoblastic osteosarcoma |
| Labrador Retriever | FS | 10 | Right proximal humerus | Osteosarcoma |
| Rottweiler | FS | 8 | Left distal tibia | Moderately-differentiated osteolytic osteosarcoma |
| Golden Retriever | FS | 6.5 | Left distal radius | Osteoblastic osteosarcoma |
| Great Pyrenes | MC | 10 | Proximal tibia | Moderately-differentiated osteosarcoma |
| Rottweiler | FS | 8 | Left proximal humerus | Osteoblastic osteosarcoma |
| Greyhound | MC | 9 | Right distal femur | Osteosarcoma |
| Rottweiler | FS | 8 | Right distal radius | Osteoblastic osteosarcoma |
| Scottish Deerhound | MC | 8 | Right distal radius | Osteoblastic osteosarcoma |
| Doberman | MC | 10 | Right distal tibia | Osteosarcoma |
| Rottweiler | MC | 8 | Right distal femur, right proximal humerus | Osteosarcoma |
| Labrador Retriever | MC | 7 | Right proximal humerus | Osteoblastic osteosarcoma |
| German Shepherd | F | 5 | Right distal tibia | Osteosarcoma with fibrosis |
| Newfoundland | FS | 5 | Left proximal humerus | Osteoblastic osteosarcoma |
| Great Dane | MC | 7 | Right distal radius | Giant cell osteosarcoma |
| Greyhound | FS | 14 | Right distal radius | Moderately-differentiated osteosarcoma |
| Greyhound | MC | 8 | Right proximal humerus | Osteoblastic osteosarcoma |
| Greyhound | FS | 11 | Left proximal humerus | Poorly-differentiated osteosarcoma |
| Greyhound | FS | 9 | Right proximal humerus | Osteoblastic osteosarcoma |
| Greyhound | MC | 6 | Right distal tibia | Poorly-differentiated osteosarcoma |
| Greyhound | FS | 8 | Distal femur | Mixed osteosarcoma |
| Greyhound | FS | 11 | Right distal femur | Mixed oteoblastic/osteolytic osteosarcoma |
| Greyhound | MC | 6 | Left distal tibia | Osteoblastic osteosarcoma |
| Greyhound | MC | 10 | Distal tibia | Osteoblastic osteosarcoma |
| Greyhound | MC | 10 | Right distal radius | Osteoblastic osteosarcoma |
| Greyhound | MC | 5 | Right proximal tibia | Osteoblastic osteosarcoma |
| Greyhound | MC | 10 | Right proximal humerus | Moderately-differentiated osteosarcoma |
| Greyhound | MC | 7 | Left distal femur | Moderately-differentiated mixed osteosarcoma |
| Greyhound | FS | 9 | Right forelimb | Moderately-differentiated osteogenic osteosarcoma |
| Doberman | MC | 6.5 | Left distal tibia | Moderately-differentiated osteoblastic osteosarcoma |
| Greyhound | MC | 6 | Right proximal humerus | Osteoblastic osteosarcoma |
| Golden Retriever | MN | 12 | Right humerus | Chondroblastic osteosarcoma |
| Greyhound | MC | 10 | Left proximal tibia | Well-differentiated osteosarcoma |
| Rottweiler | FS | 8 | Left distal tibia | Osteoblastic osteosarcoma |
| Mixed Breed | MC | 10 | Left distal ulna | Osteogenic osteosarcoma |
| Rottweiler | FS | 10 | Right proximal humerus | Moderately-differentiated mixed osteoblastic/chondroblastic osteosarcoma |
| Greyhound | MN | 11 | Left proximal humerus | Osteoblastic osteosarcoma |
| Rottweiler | MC | 5 | Left distal femur | Moderately-differentiated osteosarcoma |
| Rottweiler | MC | 4 | NR | Osteoblastic osteosarcoma |
| Rottweiler | MC | 4 | NR | Osteoblastic osteosarcoma |
| Golden Retriever | FS | 6 | Right proximal tibia | Osteoblastic osteosarcoma |
| Rottweiler | NR | NR | NR | Osteosarcoma |
| Rottweiler | M | 10 | Right distal humerus | Osteoblastic osteosarcoma |
| Rottweiler | M | 1 | Left proximal humerus | Osteoblastic osteosarcoma |
| Rottweiler | MC | 9 | Left and right distal radius | Mixed osteoblastic/fibroblastic osteosarcoma |
| Rottweiler | F | NR | Right femur | Fibroblastic osteosarcoma |
| Golden Retriever | FS | 2 | Thoracic limb | Osteoblastic osteosarcoma |
| Rottweiler | F | 10 | NR | Osteoblastic osteosarcoma |
| Golden Retriever | NR | NR | NR | Osteosarcoma |
| Rottweiler | NR | NR | NR | Osteosarcoma |
| Golden Retriever | FS | 9 | NR | Osteoblastic osteosarcoma |
| Golden Retriever | NR | NR | NR | Osteosarcoma |
| Rottweiler | NR | NR | NR | Osteosarcoma |
| Rottweiler | M | NR | NR | Osteoblastic osteosarcoma |
| Rottweiler | NR | NR | NR | Osteosarcoma |
| Rottweiler | NR | NR | NR | Osteosarcoma |
| Rottweiler | NR | NR | NR | Osteosarcoma |
| Rottweiler | FS | 12 | Left distal radius | Osteoblastic osteosarcoma |
| Rottweiler | MC | 9 | Left humerus | Chondroblastic osteosarcoma |
| Rottweiler | NR | NR | NR | Osteosarcoma |
| Golden Retriever | NR | NR | NR | Osteosarcoma |
| Golden Retriever | NR | NR | NR | Osteosarcoma |
| Golden Retriever | NR | NR | NR | Osteosarcoma |
| Golden Retriever | NR | NR | NR | Osteosarcoma |
| FS - spayed female; F - female; MC - neutered male; M - male; NR - not recorded | | | |  |
