# Supplementary figures and images for: MiR-9 is overexpressed in spontaneous canine osteosarcoma and promotes a metastatic phenotype including invasion and migration in osteoblasts and osteosarcoma cell lines
Source: BMC Cancer. 2016 Oct 10;16:784. doi: 10.1186/s12885-016-2837-5 (PMC5057229; doi:10.1186/s12885-016-2837-5)

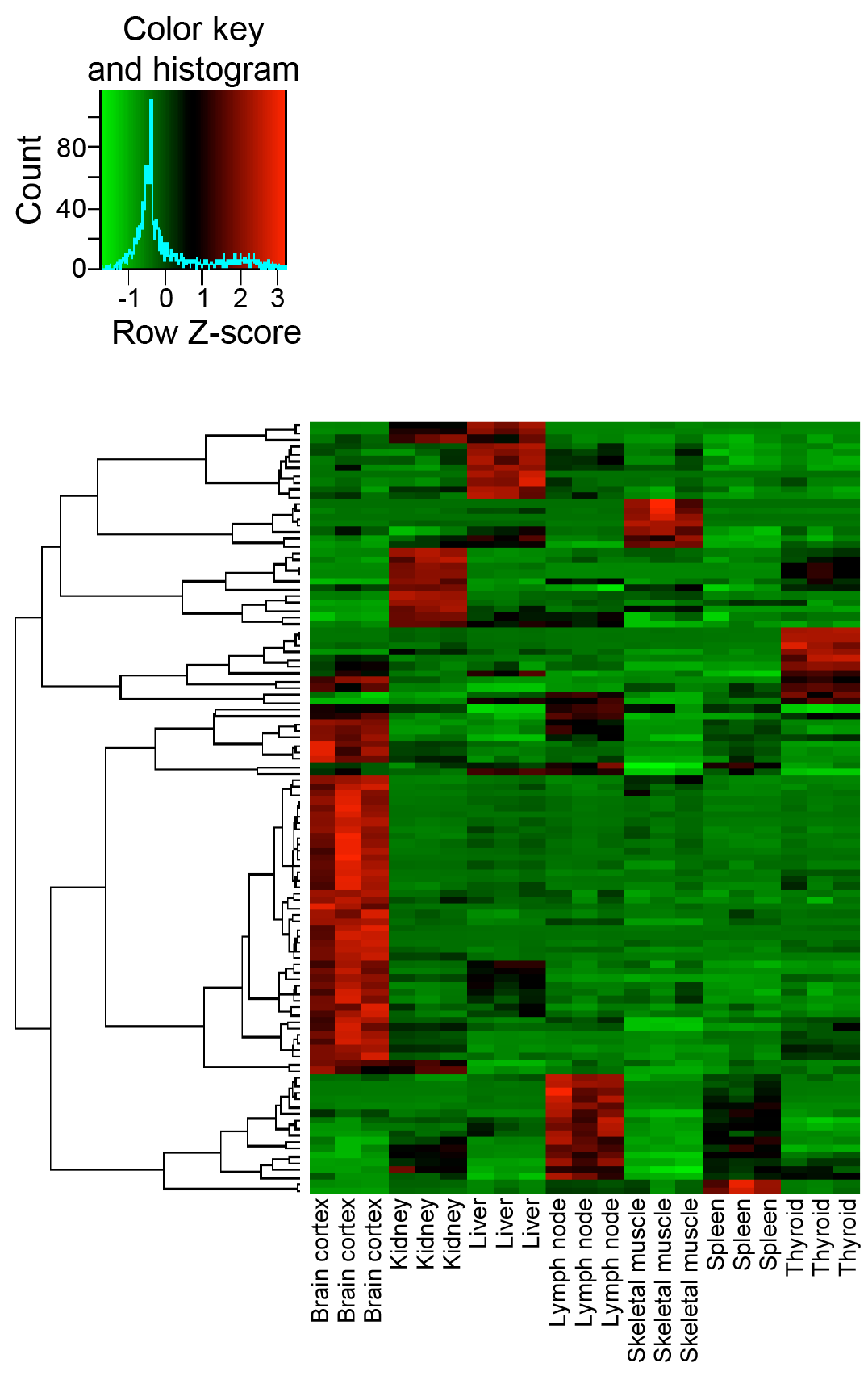

Supplement: Additional file 2: Figure S1. — MiRNA expression profiling of normal canine tissues using the nanoString nCounter platform. MicroRNA expression was evaluated in normal canine tissues (brain cortex, liver, lymph node, kidney, skeletal muscle, spleen, thyroid; N = 3 per tissue) using the Human (V2) miRNA Expression Assay CodeSet. Hierarchical clustering was performed for 110 miRNAs demonstrating unique tissue-specific expression profiles as determined by one-way ANOVA comparison test (p < 1e-06). (TIF 5836 kb) [file 12885_2016_2837_MOESM2_ESM.tif]

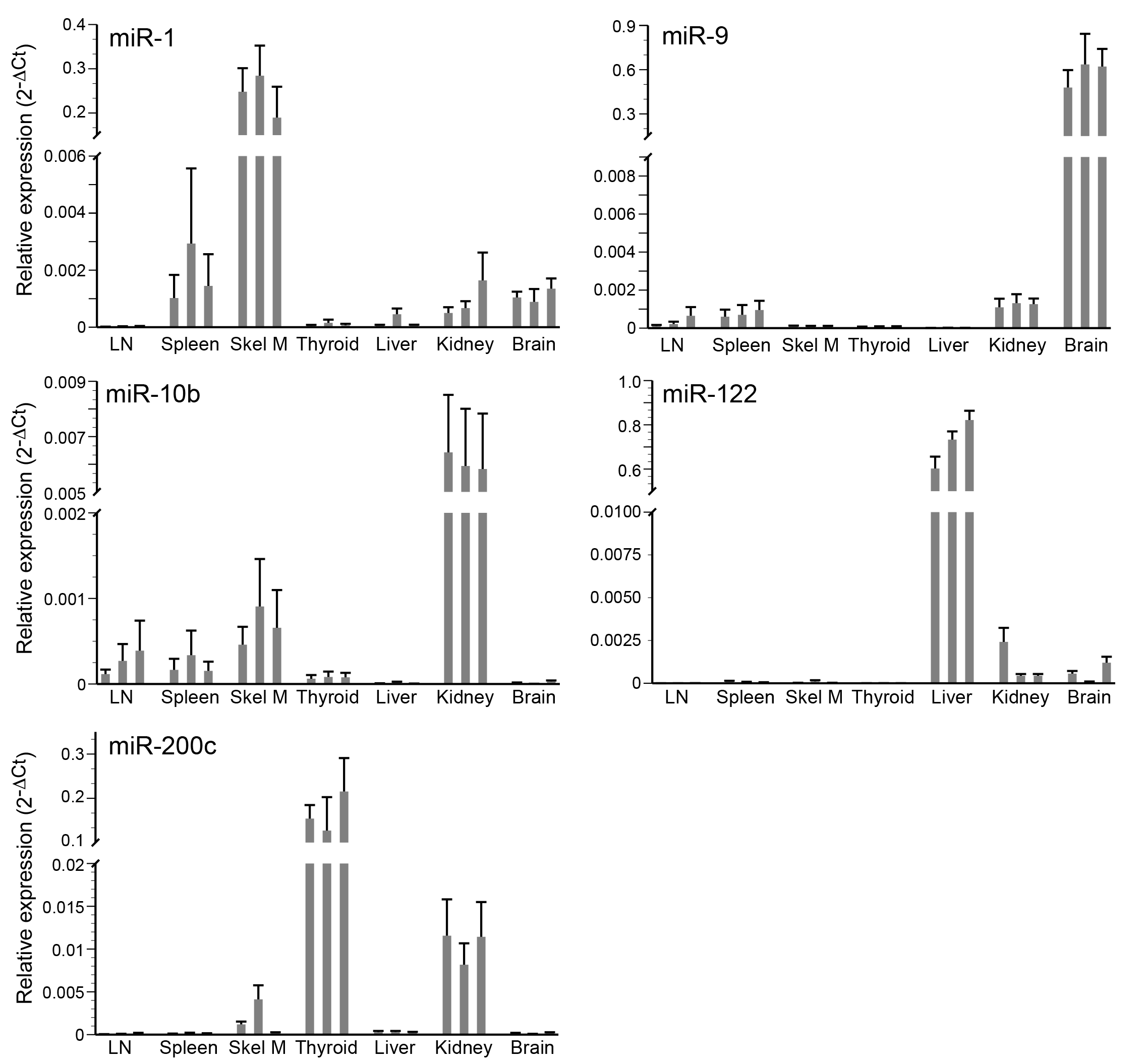

Supplement: Additional file 3: Figure S2. — Validation of nanoString profiling data in normal canine tissues. Real-time PCR was performed to independently validate changes in tissue-specific miRNA expression in normal canine tissues identified by the nanoString nCounter system. Real-time PCR confirmed differential expression of 5 representative miRNAs (miR-1, miR-9, miR-10b, miR-122, miR-200c) in normal canine tissues (brain cortex, liver, lymph node, kidney, skeletal muscle, spleen, thyroid; N = 3 per tissue) (Bars: SD. Statistical analysis: one-way ANOVA, *p < 0.05). Three independent experiments were performed and all reactions were run in triplicate. (PNG 163 kb) [file 12885_2016_2837_MOESM3_ESM.png]

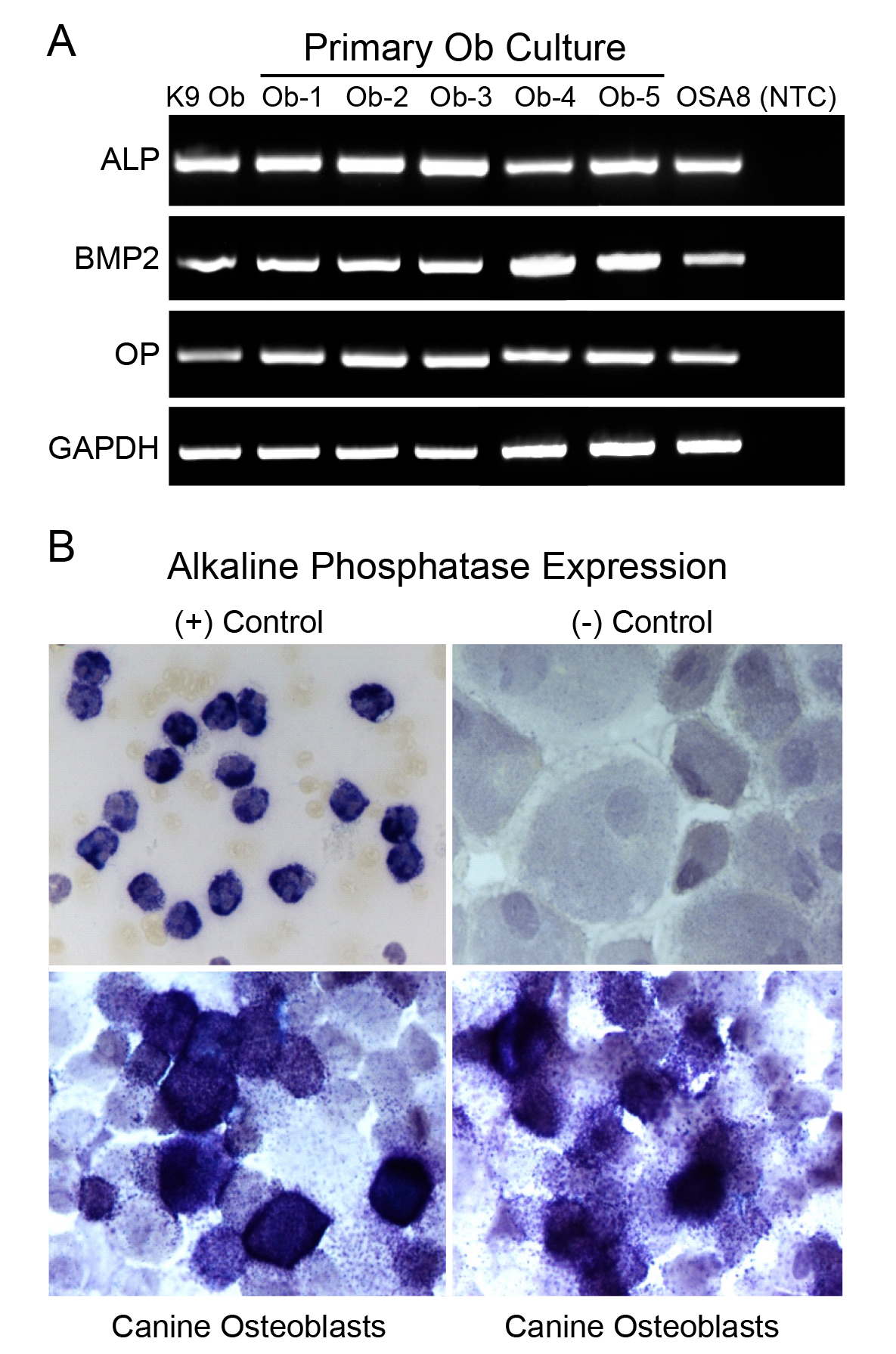

Supplement: Additional file 4: Figure S3. — Expression of bone markers in primary canine osteoblast cultures. (A) RNA was collected from five canine osteoblast (Ob) cultures established from canine patients, normal canine osteoblast cells (K9 Ob), and a canine OSA cell line (OSA8) and RT-PCR was performed for alkaline phosphatase (ALP), bone morphogenic protein-2 (BMP2), osteopontin (OP), and GAPDH. NTC = non-template control. (B) Primary canine osteoblast cultures were evaluated for ALP expression using immunocytochemistry (Sigma). Upper left panel: horse neutrophils (positive control), Upper right panel: stem cells cultured in non-differentiating conditions (negative control), Lower two panels: expression of ALP in differentiated primary canine osteoblast cultures. (TIF 9327 kb) [file 12885_2016_2837_MOESM4_ESM.tif]

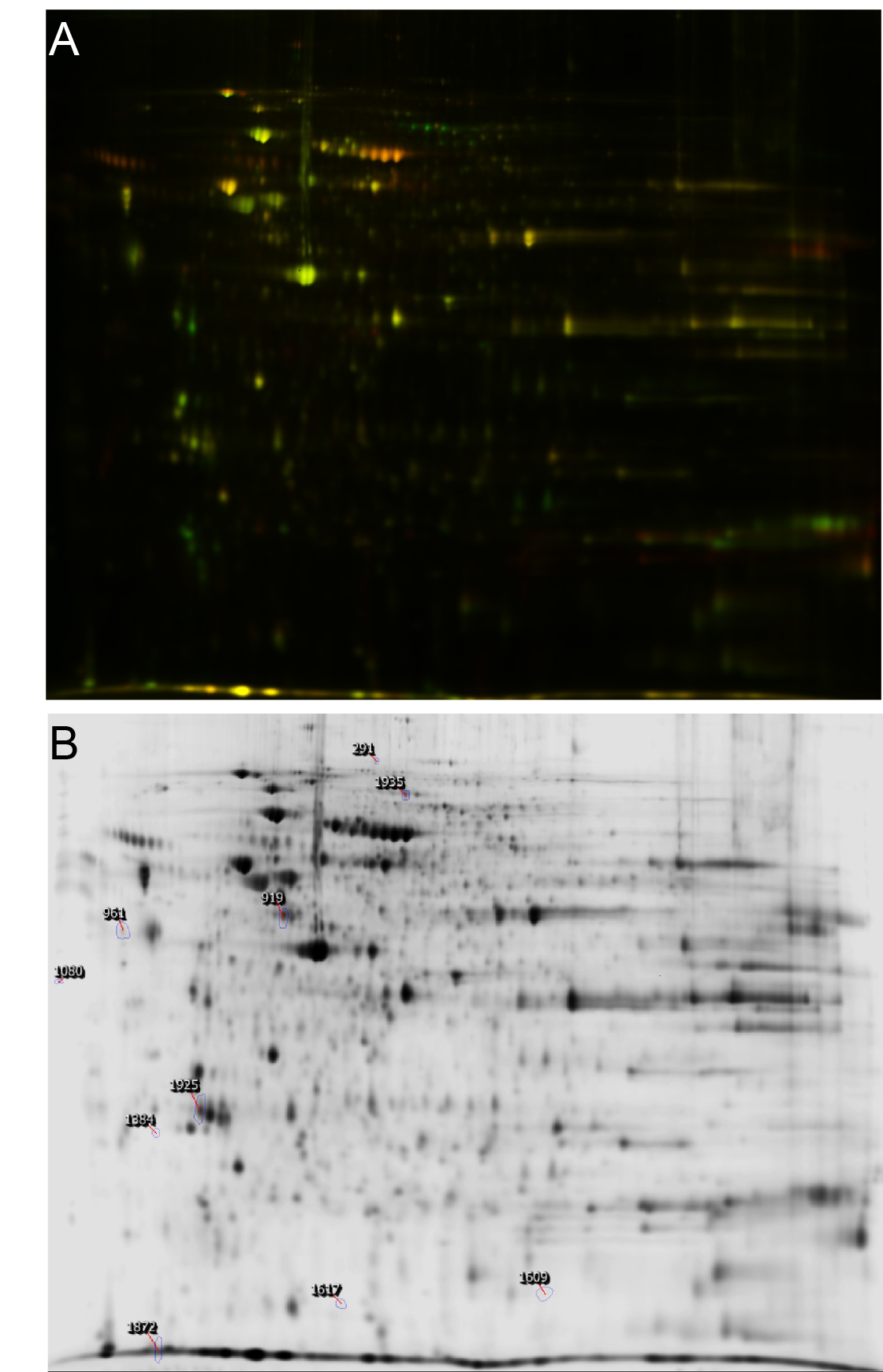

Supplement: Additional file 5: Figure S4. — 2-dimensional difference-in-gel electrophoresis (A) Representative gel image showing Cy3 (Ob-miR-9) and Cy5-labeled (Ob-EV) proteins that were isoelectric focused on pH strips (3-10), separated by size using SDS-PAGE, and visualized using a Typhoon 9400 variable mode imager. A merged image of the Cy3 and Cy5-labeled proteins is shown. Proteins with greater abundance in the miR-9-transfected sample appear green and proteins with greater abundance in the EV-transfected samples appear red. Proteins that did not change relative abundance between the two samples appear yellow. (B) Master gel image of spots exhibiting statistically significantly protein expression changes. Core protein spots of interest were excised from preparative gels stained with Lava purple general protein stain, digested with trypsin, and subject to capillary-liquid chromatography-tandem mass spectrometry analysis. The spots that were cored for subsequent protein identification are outlined and annotated (the number for each spot is the master spot number and corresponds to the Spot # listed in Table 3). (TIF 8721 kb) [file 12885_2016_2837_MOESM5_ESM.tif]
