# Supplementary material for: MiR-9 is overexpressed in spontaneous canine osteosarcoma and promotes a metastatic phenotype including invasion and migration in osteoblasts and osteosarcoma cell lines
Source: BMC Cancer. 2016 Oct 10;16:784. doi: 10.1186/s12885-016-2837-5 (PMC5057229; doi:10.1186/s12885-016-2837-5)
Supplement: Additional file 6: Table S2. — Altered gene transcripts in canine osteoblasts overexpressing miR-9 (DOCX 18 kb) [file 12885_2016_2837_MOESM6_ESM.docx]

**Table S2 Altered gene transcripts in canine osteoblasts overexpressing miR-9**

| **Downregulated transcripts** | | | | | |
| --- | --- | --- | --- | --- | --- |
|  | **FANCD2** | SHCBP1 | CDCA2 | MELK | NCAPH |
|  | C10orf90 | **KIAA0101** | FANCA | **TCF19** | E2F7 |
|  | FAM83D | MKI67 | KNTC1 | MCM2 | RACGAP1 |
|  | TK1 | GTSE1 | DSCC1 | GMNN | SHCBP1 |
|  | AURKA | SPAG5 | ERCC6L | BRCA2 | **PRND** |
|  | MAD2L1 | MYBL2 | FBXO5 | GREM2 | MAGEB18 |
|  | **STMN1** | **PKMYT1** | **SLC45A1** | **MME** | MIS18A |
|  | CDC6 | TOP2A | PARPBP | PAI-1 | **CENPA** |
|  | TICRR | BUB1 | CCDC150 | TUBA1C | CA12 |
|  | CENPW | **KIF23** | **BRCA1** | GEN1 | CENPU |
|  | CIT | **NUF2** | KNSTRN | FAM64A | PRR11 |
|  | CENPN | CDCA3 | CDC25C | DLGAP5 | ASPM |
|  | NUSAP1 | CLSPN | **DNA2** | **CDCA5** | KIF14 |
|  | **NSUN7** | DEPDC1B | PRSS53 | ORC6 | PLK4 |
|  | DCK | **RRM2** | BLM | **HMMR** | AIPL1 |
|  | SLC4A10 | **CCNF** | TPX2 | STIL | TNFRSF11B |
|  | **KIF11** | **CASC5** | SGOL1 | RAD51AP1 | **KIAA1244** |
|  | **TGFBI** | E2F1 | NOXRED1 | **NPNT** | **CA14** |
|  | **CENPE** | TACC3 | MASTL | AURKB | **OIP5** |
|  | FOXM1 | BUB1B | DKK1 | IQGAP3 | U8 |
|  | ZWINT | ANLN | **COL4A2** | DIO3 | MCM10 |
|  | CDK1 | **PRC1** | AUNIP | EXO1 | **ESCO2** |
|  | **CHAF1A** | **HAS2** | B3GALNT1 | CENPM | **NCAPG** |
|  | **SASS6** | **CEP55** | **DIAPH3** | ESPL1 | CA12 |
|  | **COL4A1** | ECT2 | **HOXB6** | TPX2 | MCM5 |
|  | CENPH | MND1 | ESCO2 | ORC1 | CDC45 |
|  | MCM3 | NEK2 | ECT2 | PLK1 |  |
|  | DSN1 | SPC25 | **PIF1** | UBE2C |  |
| **Bold indicates predicted miR-9 targets* | | | | | |
| **Upregulated transcripts** | | | | | |
|  | ACTA1 | CA2 | U1 chr36 | PTPRC  U2  U5 | 7SK chr35  U11 |
|  | VCAM1 | U12 | HIST1H4I | RNaseP_nuc | SNORA8 |
|  | EBF4 | HIST1H2AC | SNORA73 | SNORA74 | U4 chr26 |
|  | CYP4V2 | DOCK2 | ACTC | HIST2H4B | U6 |
|  | U1 chr36 | SLC6A13 | TSPAN33 | C1orf122 | SOX2OT_exon2 |
|  | NPM1 | SNORA73 | BCL11A | 7SK chr12.2 | OIP5 |
|  | ANK1 | CXCL10 | CELF2 | U2 chr35 | U8 |
|  | FAM32A | U2 chr30 | RNase_MRP | KCNE3 | U3 chr11 |
|  | U4 chr28 | SBSPON | NFATC3 | 7SK chr12.1 | U2 chr14 |
|  | U2 chr27 | 7SK chr14 | U11 | U5 | U1 chr1 |
|  | 7SK chr14 | YWHAQ | U2 chr4 | U1 chr18 | 7SK chr20 |
